# Deoxycholic acid modulates the progression of gallbladder cancer through N^6^-methyladenosine-dependent microRNA maturation

**DOI:** 10.1038/s41388-020-1349-6

**Published:** 2020-06-08

**Authors:** Ruirong Lin, Ming Zhan, Linhua Yang, Hui Wang, Hui Shen, Shuai Huang, Xince Huang, Sunwang Xu, Zijie Zhang, Weijian Li, Qiang Liu, Yongsheng Shi, Wei Chen, Jianxiu Yu, Jian Wang

**Affiliations:** 1 Department of Biliary-Pancreatic Surgery, Renji Hospital, School of Medicine, Shanghai Jiao Tong University, Shanghai, 200127 China; 2 Department of Pathology, Renji Hospital, School of Medicine, Shanghai Jiao Tong University, Shanghai, 200127 China; 3 Department of Biochemistry and Molecular Cell Biology, Shanghai Key Laboratory of Tumor Microenvironment and Inflammation, Shanghai Jiao Tong University School of Medicine, Shanghai, 200025 China; 4 Basic Clinical Research Center, Renji Hospital, School of Medicine, Shanghai Jiao Tong University, Shanghai, 200127 China

**Keywords:** Biliary tract cancer, Cell signalling, Non-coding RNAs

## Abstract

Bile acids (BAs), well-defined signaling molecules with diverse metabolic functions, play important roles in cellular processes associated with many cancers. As one of the most common BAs, deoxycholic acid (DCA) is originally synthesized in the liver, stored in the gallbladder, and processed in the gut. DCA plays crucial roles in various tumors; however, functions and molecular mechanisms of DCA in gallbladder cancer (GBC) still remain poorly characterized. Here, we analyzed human GBC samples and found that DCA was significantly downregulated in GBC, and reduced levels of DCA was associated with poor clinical outcome in patients with GBC. DCA treatment impeded tumor progression by halting cell proliferation. DCA decreased miR-92b-3p expression in an m^6^A-dependent posttranscriptional modification manner by facilitating dissociation of METTL3 from METTL3–METTL14–WTAP complex, which increased the protein level of the phosphatase and tensin homolog, a newly identified target of miR-92b-3p, and subsequently inactivated the PI3K/AKT signaling pathway. Our findings revealed that DCA might function as a tumor suppressive factor in GBC at least by interfering with miR-92b-3p maturation, and suggested that DCA treatment could provide a new therapeutic strategy for GBC.

## Introduction

Gallbladder cancer (GBC) represents almost all common malignancies of the biliary tract, with a median survival rate of 6 months [1]. Although relatively rare, it is the sixth most common gastrointestinal cancer worldwide [2]. Complete surgical resection is the only potentially curative therapy for GBC; however fewer than 10% of patients are considered surgical candidates owing to the advanced stage of disease in the majority, resulting in a poor clinical outcome [3]. At present, the chemotherapy with cisplatin plus gemcitabine has been proposed as an appropriate choice for patients with advanced GBC [4, 5]. Despite this, the prognosis for the patients undergoing combination chemotherapy treatment is dismal, with survival estimation of <1 year. Although GBC is very aggressive, knowledge of its etiology remains limited. Several factors may contribute to the development of GBC, such as genetic predisposition, geographic variation, and chronic inflammation [6]. In brief, lacks of a biomarker for early diagnosis and effective chemotherapy with a defined target for GBC are still a major hindrance for treatment, thus identifying novel targets both for diagnosis and treatment need to be urgently resolved.

Bile acids (BAs), stored in the gallbladder, are metabolic molecules with diverse endocrine and paracrine functions [7–11]. At present BAs and BA receptors have emerged as targets for the treatment of metabolic diseases such as obesity, diabetes, steatohepatitis, and atherosclerosis [9–16]. Intriguingly, BAs have contradictory roles in different cell types. Various BAs exert proliferative effects on a plethora of cell types, including hepatocytes, cholangiocytes, and intestinal epithelial cells [17, 18], whereas some BAs such as taurocholic acid and tauroursodeoxycholic acid (TUDCA) promote apoptosis in hepatocytes [19–21]. Furthermore, in contrast to its effect on hepatocytes, TUDCA induces the proliferation of cholangiocytes in vitro and in vivo [21, 22]. The vast heterogeneity of BAs within cell types crucially complicates the assessment of potential diagnosis and therapy. Whether BAs exerting protective or toxic effects may depend on their biochemical properties and concentration, as well as the BA receptors expressed in the respective cell types [13]. Although intensive efforts have been made to explore the roles of BAs in various cell types, little focus has been paid to the effects of BAs on GBC.

Recently N^6^-methyladenosine (m^6^A) as the important modification of RNA has attracted considerable interests since it plays a central role in eukaryotes [23–25]. The m^6^A modification of RNA is dynamic and reversible in eukaryotes with the involvement of two predominant catalytic proteins. The core methyltransferase complex consists of methyltransferase-like 3 (METTL3) [26], methyltransferase-like 14 (METTL14) [27], and Wilms tumor 1-associated protein (WTAP) [28], which transfers with the methyl group during m^6^A modification. In contrast, fat mass and obesity-associated protein [29] and AlkB homolog 5 [30] serve as demethylases. The homeostasis of m^6^A modification is mainly determined by the regulation between m^6^A methyltransferases and demethylases. A growing body of evidence indicates the crucial roles of changed m^6^A levels in diverse biological functions in eukaryotes, such as transcription splicing [31, 32], posttranscriptional regulation [33], nuclear RNA export [34], posttranslational regulation [35], as well as cell fate determination [36, 37]. Perturbations of the regulatory machinery of m^6^A methylation are associated with several human diseases or cancers [30, 31, 36]. However, the molecular mechanisms underlying the regulation of m^6^A methylation status in cancers, especially in GBC, remain poorly investigated.

In this study, we showed that deoxycholic acid (DCA) is downregulated in GBC and controls tumor progression by suppressing the maturation of microRNAs (miRNAs) in an m^6^A-dependent transcriptional modification manner. In line with these observations, the DCA treatment inhibited tumor growth of xenograft GBC model in mice. Furthermore, the DCA level was significantly correlated with favorable clinical outcomes in patients with GBC. Taken together, our findings suggested that DCA treatment might be a therapeutic strategy for GBC.

## Results

### Analyses of serum DCA levels in human GBC samples

**Fig. 1:**
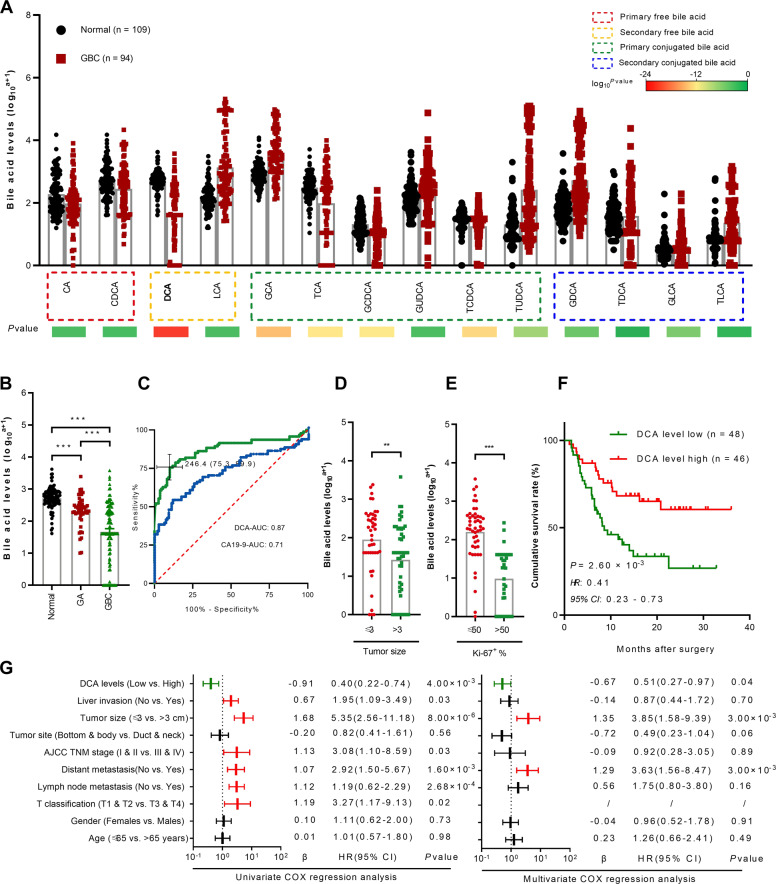
Reduced DCA is associated with poor clinical outcome in human gallbladder cancers. **a** Serum levels of primary free bile acid, secondary free bile acid, primary conjugated bile acid, and secondary conjugated bile acid in normal individuals and GBC patients (means ± SEM, *n* = 109 vs. 94; *p < 0.05). **b** Serum DCA levels in normal individuals compared with GA and GBC patients. **c** Receiver operating characteristic (ROC) curves of serum DCA and carbohydrate antigen 19-9 (CA19-9) for the presence of GBC with the area under the ROC curve (AUROC). Measurement of serum DCA level from GBC patient in different tumor size (**d**) and Ki-67 (**e**) expression. **f** Kaplan–Meier survival curves of overall survival according to serum DCA level by using the online bioinformatics tool Kaplan–Meier plotter (*p < 0.05). **g** Multivariable analysis of hazard ratios for overall survival in GBC by using the logistic regression model. CA cholic acid, CDCA chenodeoxycholic acid, DCA deoxycholic acid, LCA lithocholic acid, GCA glycocholic acid, TCA taurocholic, GCDCA glycochenodeoxycholic acid, GUDCA glycoursodeoxycholic acid, TCDCA taurochenodeoxycholic acid, TUDCA tauroursodeoxycholic acid, GDCA glycodeoxycholic acid, TDCA taurodeoxycholic acid, GLCA glycolithocholic acid, TLCA taurolithocholic acid.

Accumulating evidence indicates that the disruption of BA homeostasis is the main hallmarks of diseases, by favoring cell autonomous and nonautonomous processes, which may modulate tumor growth [38]. Considering that BAs are re-absorbed into the blood, the levels of primary free, secondary free, primary conjugated and secondary conjugated BAs were measured in the serum samples collected from normal individuals and patients with GBC. Unlike in normal individuals, most serum BA levels were significantly increased in patients with GBC (Fig. 1a), which represented significant disruption of BAs homeostasis and might affect tumor initiation and progression. In contrast, DCA, a secondary free BA, was markedly decreased in patients with GBC. Furthermore, gallbladder adenoma (GA), a gallbladder tumor with less aggressive phenotype compared with that of GBC, showed median DCA serum level (Fig. 1b), indicating that the level of DCA downregulation was depended on the cancer phenotype. These findings suggested that DCA might be a novel diagnostic biomarker for GBC.

To explore the possibility of using serum DCA as a diagnostic biomarker for patients with GBC, we evaluated receiver operating characteristics (ROC) for diagnostic ability. Given that carbohydrate antigen 19-9 (CA19-9) has been widely used in the diagnosis of gastrointestinal tumors including GBC, we selected it as a standard diagnostic biomarker. The area under the ROC curve of serum DCA level was 0.87, which was higher than that of CA19-9 with 0.71 (Fig. 1c). Furthermore, lower DCA serum level indicated more aggressive phenotype of GBC, including larger tumor size and stronger Ki-67 staining (Fig. 1d, e). Thus, the serum DCA level might be a potential diagnostic biomarker for GBC.

Furthermore, patients with GBC having decreased DCA levels had poorer overall survival (*n* = 94, *p* < 0.001, log-rank test; Fig. 1f). In addition, univariate Cox regression analysis revealed that liver invasion, tumor size, TNM stage, distant metastasis, lymph node metastasis, T classification, and serum DCA level were substantially associated with survival in patients with GBC (Fig. 1g), and multivariate Cox regression analysis showed that DCA was an independent predictive marker for the prognosis of patients with GBC (HR = 0.51, 95% CI (0.27–0.97); Fig. 1g; Supplementary Table S1). Taken together, our findings showed that DCA was downregulated in human GBC and was associated with poor clinical outcome, prompting us to identify DCA as a potential biomarker for GBC.

### DCA exerts tumor suppressive activities in GBC cells

**Fig. 2:**
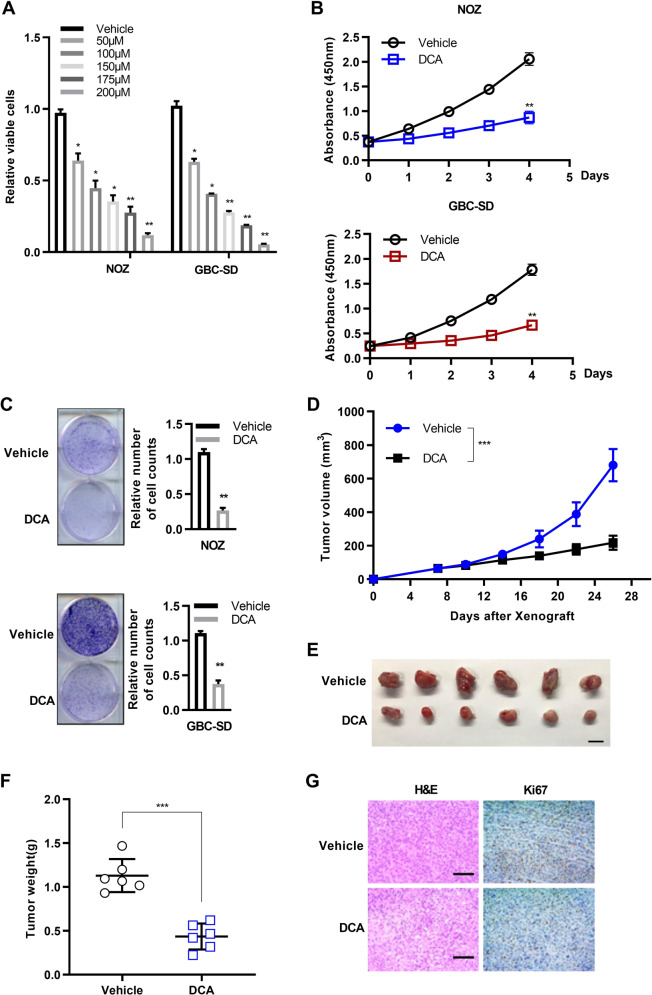
DCA impairs gallbladder cancer cell proliferation in vitro and in vivo. **a** DCA treatment was significantly cytotoxic to human gallbladder cancer cells (72 h). One-way analysis of variance (ANOVA) (means ± SEM, *p < 0.05). **b** Cell proliferation assay of gallbladder cancer cells challenged with vehicle and DCA. (*n* = 3, in triplicate; two-way ANOVA, comparisons at days 4 are shown). **c** Colony formation assay of vehicle- and DCA-treated gallbladder cancer cells. Representative images to corresponding experiments, and the graph exhibits the quantification of colony numbers. The relative cell numbers of vehicle were arbitrarily set to 1 (*n* = 3 independent experiments, in triplicate; Mann–Whitney test). **d** Follow-up of tumor growth after nude mice were subcutaneously injected with GBC-SD cancer cells, following feeding of food with or without DCA. (*n* = 6 per group; two-way ANOVA, comparisons at day 26 are shown; ***p < 0.001). **e** Excised xenograft tumors 4 weeks from **d**. Scale bars represent 10 mm. **f** Tumor weight in d are shown (**p < 0.01). **g** Representative images of Ki-67 immunohistochemical (IHC) staining in **d** from two groups are shown. Scale bars represent 100 μm.

Recent evidence shows that some BAs are accumulated in certain cancers to suppress tumor proliferation [39], suggesting that BAs may serve as tumor suppressors. Since that gallbladder epithelium was directly infiltrated in gallbladder BAs, which was closely associated with serum BAs [10], we adopted the concentration range of DCA in gallbladder BAs for the further study. Due to difficult to obtain gallbladder BAs in healthy individuals, we have just detected it in several healthy individuals derived from liver transplantation donors (*n* = 18, ***p < 0.001; Supplementary Fig. S1A). Then, we tested the cell viability at DCA concentration from nM to μM range in three human GBC cell lines (Supplementary Fig. S1B). We found that the cell viability of all three GBC cells was affected in μM range, which was quite consistent with that reported in the literatures for other BAs [18, 40–42]. To determine whether DCA impacts GBC progression, we assessed the biological behaviors of two human GBC cell lines NOZ and GBC-SD by the treatment with different concentrations of DCA in vitro. DCA treatment significantly reduced the viability of GBC cells in a dosage-dependent manner (Fig. 2a). Next, we investigated whether DCA affects GBC cell proliferation. The results of proliferation assays showed that DCA treatment remarkably inhibited proliferation of both NOZ and GBC-SD cells (Fig. 2b), indicating the potential suppressive effect of DCA on GBC tumor cell growth. Indeed, the colony formation assays showed that the colony numbers of both NOZ and GBC-SD cells were significantly reduced by DCA treatment (Fig. 2c). Further, we investigated the effects of DCA on GBC xenografted tumor growth in nude mice. Mice were first subcutaneously injected with GBC cancer cells, followed by feeding a diet containing 0.1% DCA or equal amount of vehicle. Compared with the control group (vehicle), the growth rates (Fig. 2d, e) and weights (Fig. 2f) of excised tumors were significantly reduced in the DCA treatment group. In agreement with these observations, histopathological analyses of tumors derived from different mice revealed lower proliferation rates in the DCA treatment than in the vehicle control groups, as shown by Ki-67 immunostaining (Fig. 2g). To determine whether DCA affects non-transformed cells, H69, a well-established non-malignant cholangiocyte (originate from bile duct epithelium), were treated with DCA. The result showed that the concentration of DCA we used in this assay had no effect on H69 (Supplementary Fig. S1C). On the basis of its origin in BAs process, we also tested the effect of GDCA and TDCA (two conjugated forms of DCA) on GBC cells. These two BAs showed similar effect on GBC cells, but at the higher concentration than DCA (Supplementary Fig. S1D, E). Taken together, above results in vitro and in vivo demonstrated that DCA had a tumor suppressive function in GBC.

### DCA reduces the expression of miR-92b-3p in GBC cells

**Fig. 3:**
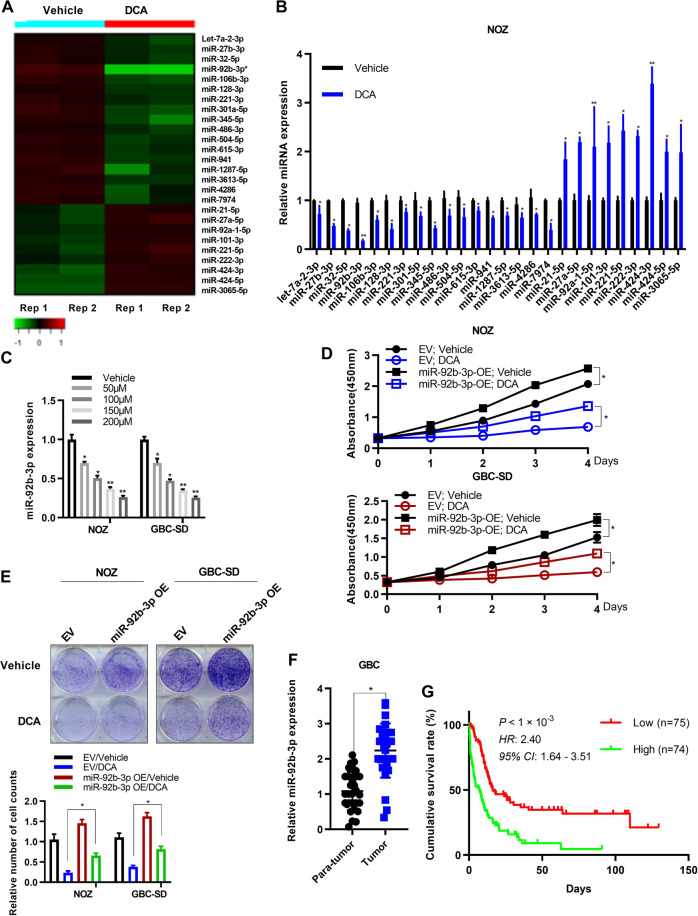
DCA treatment regulates miR-92b-3p expression in gallbladder cancer cells. **a** Heatmap representation of significantly differentially expressed miRNAs (*p* < 0.05; fold change, >2 or <0.5) detected using miRNA sequence in NOZ cells treated with DCA (50 μM) or equal amount of vehicle as control. **b** Expression levels of indicated miRNAs in NOZ cells treated with DCA or vehicle as in **a**. Results represent means ± standard derivation (SD) from three independent measurements. **c** The decreased expression of miR-92b-3p in NOZ and GBC-SD cells in a DCA dose-dependent manner (0, 50, 100, 150, and 200 μM). Data represent mean ± SD from three independent experiments. **d** Cell proliferation assay of gallbladder cancer cells overexpressing miR-92b-3p or empty vector challenged with vehicle and DCA. (*n* = 3, in triplicate; two-way ANOVA, comparisons at days 4 are shown). **e** Colony formation assay of vehicle- and DCA-treated gallbladder cancer cells overexpressing miR-92b-3p or empty vector. Representative images for corresponding experiments, and the graph exhibits the quantification of colony numbers. The relative cell numbers of vehicle were arbitrarily set to 1 (*n* = 3 independent experiments, in triplicate; Mann–Whitney test). **f** miR-92b-3p RNA levels were quantified in 38 pairs of GBC tissues and adjacent normal tissues by using qPCR (two-way analysis of variance (ANOVA); ***p < 0.001). **g** Kaplan–Meier survival curves of overall survival according to miR-92b-3p level (*p < 0.05).

Next, we explored the mechanisms downstream of DCA underlying its tumor suppressive functions. Over the past decade, miRNAs, a class of non-coding RNAs, have emerged as crucial regulatory molecules in cancers [43]. Therefore, we performed high-through miRNA-seq, and found that 26 miRNAs were significantly differentially expressed in NOZ cells treated with DCA, compared with those in cells treated with vehicle (*p* < 0.05, Student *t* test). Analysis of the results showed that 17 miRNAs were downregulated (fold change, <0.5; Fig. 3a), whereas a smaller subset of miRNAs were upregulated (fold change, >2) after DCA treatment (Fig. 3a). In addition, the results were further verified using quantitative real-time PCR (qRT-PCR) analysis in both NOZ and GBC-SD cells treated as described above (Fig. 3b; Supplementary Fig. S2A). Among the downregulated miRNAs, the most significantly downregulated was miR-92-3p (fold change = 0.125, *p* < 0.001, Student *t* test). We also confirmed that the expressions of miR-92b-3p were repressed in a DCA dosage-dependent manner in GBC cells (Fig. 3c).

To further explore the function of miR-92b-3p in GBC cells, we established NOZ and GBC-SD cell lines stably overexpressing miR-92b-3p (Supplementary Fig. S2B, C) and found that miR-92b-3p substantially enhanced cell proliferation. Furthermore, the ectopic expression of miR-92b-3p partly attenuated the DCA-mediated inhibition of tumor growth (Fig. 3d, e). Moreover, we analyzed the expression levels of miR-92b-3p in surgically removed para-tumor and tumor GBC tissue samples and found that miR-92b-3p expressions were higher in tumor tissues than in para-tumor tissues (*n* = 38, *p* < 0.01; Fig. 3f). These findings suggested that miR-92b-3p might be involved in the different stages of tumor progression and could contribute to poor clinical outcome.

To confirm our hypothesis, we investigated whether the expression of miR-92b-3p was closely correlated to the prognosis of GBC by using the database of the Cancer Genome Atlas (TCGA). However, no relationship was found between miRNA expression and GBC survival time in the TCGA, possibly because of the small sample size. Nonetheless, we found that, in liver hepatocellular carcinoma, another classic phenotype of hepatobiliary tumor, high expression of miR-92b-3p was significantly associated with shorter survival time (Supplementary Fig. S2D). Thus, we measured the expression of miR-92b-3p in GBC tissue samples collected from our hospital cancer centers (Supplementary Table S2). Kaplan–Meier plots showed that patients with GBC having overexpression of miR-92b-3p (<median) had shorter overall survival time than those with low level of miR-92b-3p (<median; Fig. 3g). Furthermore, measurement of the other changed miRNAs induced by DCA in surgically removed para-tumor and tumor GBC tissue samples showed that let-7a-2-3p level was lower in tumor tissues than that in para-tumor tissues, while miR-615-3p and miR-3065-5p levels were higher in tumor tissues than those in para-tumor tissues (Supplementary Fig. S2E). However, these miRNAs had no effect on GBC patient survival (Supplementary Fig. S2F). These data suggested that miR-92b-3p was a novel oncogenic factor for GBC with dismal clinical outcome, and DCA might function as a tumor suppressive factor by controlling miR-92b-3p expression in GBC cells.

### MiR-92b-3p targets PTEN to inhibit PI3K/AKT signaling

**Fig. 4:**
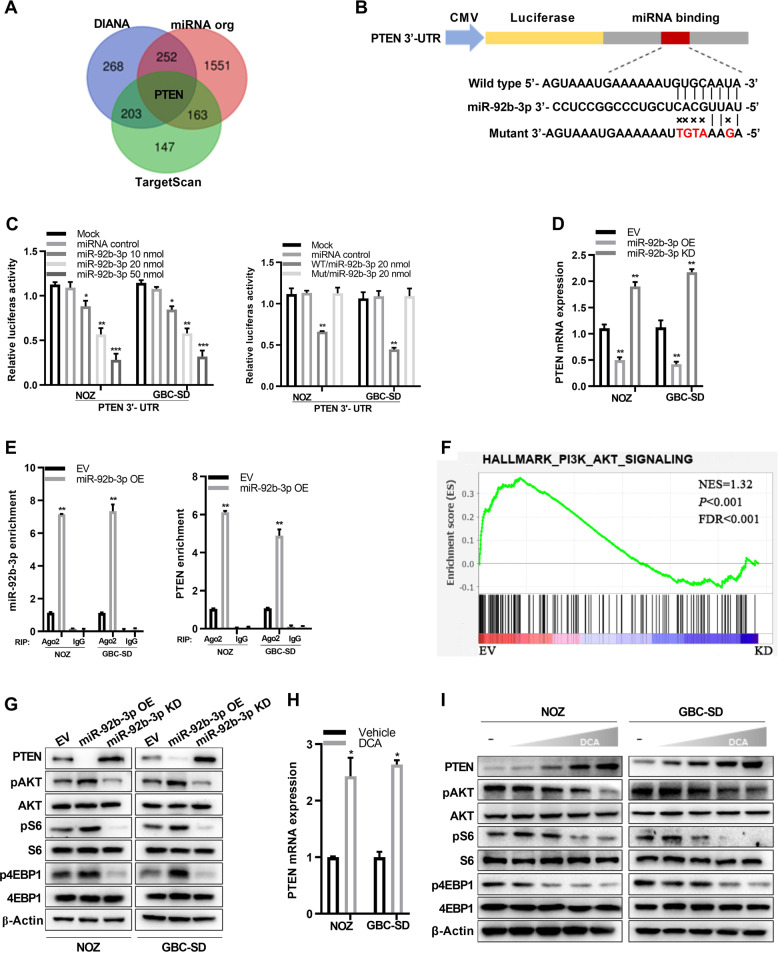
DCA-mediated miR-92b-3p reduction impedes PI3K/AKT signaling via up-regulation of PTEN. **a** Venn diagrams present potential target genes of miR-92b-3p in three databases. Among the genes overlapped in the three databases, PTEN was the highest confidence target for miR-92b-3p. **b** A schematic drawing showing the possible binding sites of miR-92b-3p within the PTEN 3′-UTR and the corresponding site-specific mutations. **c** Relative reporter gene activity of vector containing PTEN 3′-UTR in NOZ and GBC-SD cells co-transfected with increasing amounts (10, 20, and 50 pmol) of miR-92b-3p mimic. Relative reporter gene activity of vector with PTEN 3′-UTR or mutant counterparts in NOZ and GBC-SD cells in the presence of 20 pmol miR-92b-3p mimic. **d** Stable overexpression or knockdown of miR-92b-3p affects the levels of PTEN mRNA. Data shown are means ± SD from three representative independent experiments. **e** RNA levels of miR-92b-3p and PTEN in immunoprecipitates are shown as fold enrichment in AGo2 relative to those in IgG. Data shown are means ± SD from three representative independent experiments. **f** PI3K/AKT signaling in NOZ cells stably knockdown of miR-92b-3p. **g** Immunoblotting analysis for PTEN protein level, as well as AKT (Ser473), 70S6K (Thr389), and eIF4EBP1 (phospho T37) phosphorylation in NOZ and GBC-SD cells stably overexpressing or knockdown of miR-92b-3p. **h** DCA affects the expression of PTEN mRNA in NOZ and GBC-SD cells. Data shown are means ± SD from three representative independent experiments. **i** Immunoblotting analysis for PTEN protein level, as well as AKT (Ser473), 70S6K (Thr389), and eIF4EBP1 (phospho T37) phosphorylation in NOZ and GBC-SD cells treated with DCA.

To explore the potential miR-92b-3p target genes, we used three publicly available algorithms to get the Venn diagram (Fig. 4a). The result showed that phosphatase and tensin homolog (PTEN), a well-defined tumor suppressor gene, may be the potential candidate (Fig. 4b). To further confirm this, we performed the luciferase reporter assay by using the vectors containing the PTEN 3′-UTR fragments with the miR-92b-3p mimic. Compared with those in the control, miR-92b-3p showed dosage-dependent inhibition in the luciferase activity for the vector containing the PTEN 3′-UTR (WT) in both NOZ and GBC-SD cells (Fig. 4c, left panel). However, the luciferase reporter activity was not affected in the vector containing mutated PTEN 3′-UTR, in which the miR-92b-3p-binding sites were mutated (Fig. 4c, right panel). Moreover, PTEN mRNA levels were decreased by miR-92b-3p overexpression while remarkably increased by miR-92b-3p knockdown in NOZ and GBC-SD cells (Fig. 4d). We also conducted RNA immunoprecipitation (RIP) by using an antibody against Argonaute 2 (AGO2), followed by qRT-PCR. We found that both miR-92b-3p and PTEN mRNA were significantly enriched in the miRNA–RISC complex in NOZ and GBC-SD cells overexpressing miR-92b-3p (Fig. 4e). Taken together, these results suggested that PTEN was the true miR-92b-3p targeting gene.

To further determine the biological role of miR-92b-3p in GBC cells, we performed a global RNA expression analysis by using NOZ cells stably knockdown miR-92b-3p or an empty vector. As expected, the PI3K/AKT pathway was remarkably enriched in the gene set enrichment analysis plots, which was ranked as the top-ranked signaling pathway (Fig. 4f; Supplementary Fig. S3A). These results were verified using qRT-PCR analysis both in NOZ and GBC-SD cells (Supplementary Fig. S3B). Next, we determined the effects of miR-92b-3p on PI3K/AKT signaling by using immunoblotting. The protein levels of p-AKT (Ser473), p-70S6K (Thr389), and p-eIF4EBP1 (phospho T37) were significantly decreased by knockdown of miR-92b-3p, whereas their levels were substantially increased by overexpression of miR-92b-3p in both NOZ and GBC-SD cells (Fig. 4g). These data suggested that miR-92b-3p served as an upstream activator of PI3K/AKT signaling by inhibiting PTEN expression. Moreover, we showed that PTEN mRNA (Fig. 4h) and protein (Fig. 4i) levels were significantly elevated in GBC cells when treated with DCA, which downregulated expression of miR-92b-3p (Fig. 3a–c). Consistent with above findings, DCA treatment substantially decreased PI3K/AKT signaling in NOZ and GBC-SD cells (Fig. 4i). To investigate whether DCA-mediated alteration of PI3K/AKT signaling by miR-92b-3p is specific to GBC, we detected this in other cancer cell lines. We found that there were not significant changes in both miR-92b-3p (Supplementary Fig. S3C) and PTEN (Supplementary Fig. S3D) levels in SMMC-7721, PANC-1, HepG2, H1299, and LNCaP under treatment with DCA, which suggested that this pathway was not affected in these cancer cell lines by DCA. Owing its role in metabolism, the receptor for BAs has been intensively studied in recent years. Among these receptor, FXR and GPBAR1, that high affinity binding DCA, were identified as DCA receptor. Thus, we knocked down the FXR and GPBAR1 in GBC cell lines, and detected the effect of DCA on miR-92b-3p expression. We found that DCA-mediated alteration of miR-92b-3p were independent on FXR and GPBAR1 (Supplementary Fig. S3E, F). Since little is known about BAs as a metabolite, its role in pathways in cellular processes is worth taking consideration in future study. Collectively, these results demonstrated that DCA-induced downregulation of miR-92b-3p inhibited oncogenic PI3K/AKT signaling by elevating PTEN in gallbladder cancer.

### Site-specific m^6^A is associated with DCA transcriptional repression of primary miR-92b

**Fig. 5:**
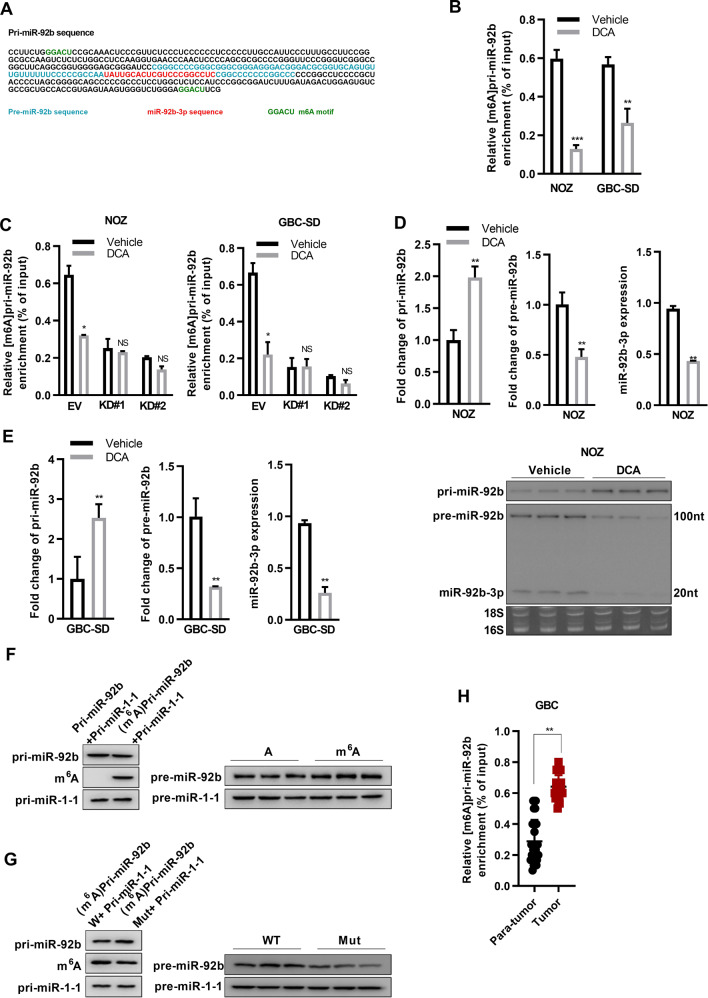
DCA induces the reduced m^6^A peaks of specific motifs in pri-miR-92b to suppress miRNA processes. **a** The sequences of pre-miR-92b, miR-92b-3p, and the potential m^6^A motif (GGACU) splicing site are highlighted with different colors. [m^6^A] pri-miR-92 was detected using immunoprecipitation, followed by qRT-PCR analysis, in NOZ (**b**) and GBC-SD (**c**) with or without the knock down of METTL3 in cells treated with DCA. (***p < 0.001 by Wilcoxon rank-sum test). Northern blot detection of the levels of pri-miR-92b, pre-miR-92b, and miR-92b-3p in NOZ (**d**) and GBC-SD (**e**) challenged with DCA or vehicle. 28S and 18S rRNAs were used as loading controls. **f** Northern blot showing initial materials (left) and the levels of sequent pre-miR-92b and pre-miR-1-1 (right) after the reaction. **g** Northern blot detection of initial materials with the putative METTL3-catalysing motif in pri-miR-92b or compartment mutant vector (left) and the levels of resultant pre-miR-92b and pre-miR-1-1 (right) in the reaction. **h** [m^6^A] pri-miR-92b was detected using immunoprecipitation, followed by qRT-PCR analysis, in GBC tumor tissue and adjacent normal tissue samples (***p < 0.001 by Wilcoxon rank-sum test).

Since m^6^A methylation has been reported to affect miRNA maturation [44, 45], we wanted to investigate whether DCA treatment alters the m^6^A methylation level of pri-miR-92b. First, we located the potential m^6^A site on pri-miR-92b by analyzing the genome sequence and found an m^6^A motification site within the pri-miR-92b (Fig. 5a). Thus, we then investigated whether DCA treatment mediates the m^6^A level of pri-miR-92b in GBC cells, by conducting a methylation RIP (MeRIP) assay with anti-m^6^A antibody. The followed qRT-PCR results showed that the m^6^A levels of pri-miR-92b were significantly lower in cells treated with DCA than those in cells without DCA treatment (Fig. 5b). Furthermore, knockdown of METTL3 (Supplementary Fig. S4A), which is a well-defined core methylation modification writer for m^6^A [46], abolished the alterations of pri-miR-92b m^6^A methylation levels mediated by DCA treatment (Fig. 5c). These results suggested that DCA mediated the alteration of m^6^A methylation of pri-miR-92b was mainly dependent on METTL3.

Next, we evaluated the expression levels of pri-miR-92b, pre-miR-92b, and miR-92b-3p in NOZ and GBC-SD cells treated with or without DCA. We found that the level of pri-miR-92b was significantly higher in cells treated with DCA than that in cells with vehicle; on the contrary, both pre-miR-92b and miR-92b-3p levels were lower after DCA treatment (Fig. 5d, e; Supplementary Fig. S4B). To support the hypothesis of m^6^A methylation regulating miR-92b-3p maturation, we conducted in vitro RNA processing assays. The pri-miR-92b was in vitro transcribed with nucleotide m^6^A or A, and then incubated with whole lysates of HEK293T cells overexpressing the miRNA processing enzymes DROSHA and DGCR8. Compared with the unmethylated counterpart, m^6^A-modified pri-miR-92b showed more efficient rate to process to pre-miR-92b and miR-92b-3p (Fig. 5f). Consistent with the notion that site-specific m^6^A is associated with pri-miR-92b methylation, the progression rate of pri-miR-92b to its mature form was substantially decreased when the putative METTL3-catalysing motif in pri-miR-92b was mutant (Fig. 5g). Since miR-92b-3p affected GBC cell line proliferation (Fig. 3d, e), m^6^A methylation of pri-miR-92b could be closely associated with GBC progression. Next, we performed m^6^A-specific RIP, followed qRT-PCR assay to determine the m^6^A methylation level of pri-miR-92b in GBC tumor tissue and adjacent normal tissue samples. The results showed that the level of pri-miR-92b m^6^A methylation was significantly higher in GBC tumor tissue samples than that in the adjacent normal tissue samples (Fig. 5h). Taken together, these in vitro and in vivo results demonstrated a suppressive role of DCA in miR-92b-3p maturation by causing a decrease in the site-specific m^6^A methylation of pri-miR-92b in GBC cells.

### DCA disrupts the METTL3–METTL14–WTAP complex by binding to METTL3

**Fig. 6:**
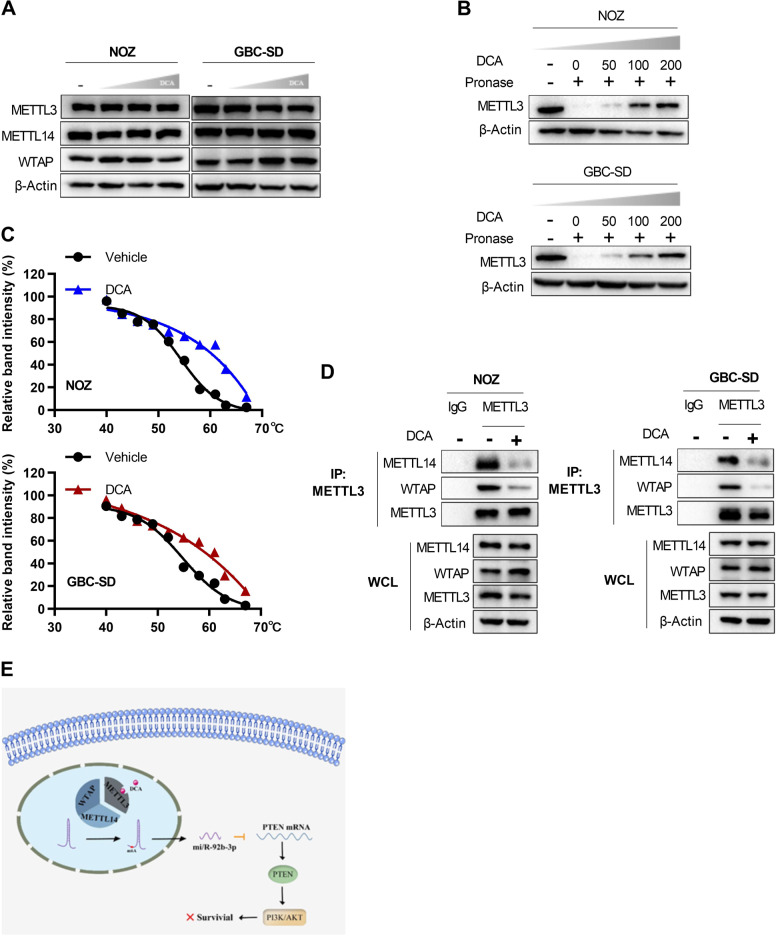
DCA disrupts the METTL3–METTL14–WATP complex through binding to METTL3. **a** METTL3, METTL14, and WTAP protein levels in NOZ and GBC-SD cells treated with DCA in an increased dosage. **b** DARTS assays show the direct binding between DCA and METTL3. **c** Identification of the binding affinity of DCA and METTL3 by using CETSAs. **d** Interactions between METTL3 and METTL14 or WTAP in the presence and absence of DCA were determined using co-immunoprecipitation experiments. **e** Schematic illustration of the dissociation of METTL3–METTL14–WTAP complex by DCA leads to the decline of miR-92b-3p by affecting [m6A] pri-miR-92b-mediated processes.

We then investigated the molecular mechanism by which DCA regulates m^6^A modification of pri-miR-92b. Accumulating evidence has indicated that the METTL3–METTL14–WTAP complex plays a crucial role in m^6^A deposition in mammalian nuclear RNAs [27, 47], thus we investigated the impact of DCA on the expressions of these three genes. However, no change in the mRNA and protein levels of these genes including METTL3, METTL14, and WTAP was observed when GBC cells were treated with DCA (Fig. 6a; Supplementary Fig. S4C, D), which indicated that DCA might function probably by destroying the METTL3–METTL14–WTAP complex other than stimulating their expressions. We reckoned that DCA might directly bind to one component (e.g., METTL3) of the complex thus to interfere the complex formation. To confirm this, we performed two types of biochemical assays. The drug affinity responsive target stability (DARTS) assays were conducted to show the direct binding between METTL3 protein and DCA in vitro, which inhibited its degradation through proteinase in a dosage-dependent manner in both NOZ and GBC-SD cells (Fig. 6b). Moreover, the cellular thermal shift assays (CETSAs) were also performed, and obvious shifts in METTL3 melting curve were observed in the presence of DCA in the CETSAs, suggesting the direct binding of METTL3 with DCA inside the cells (Fig. 6c; Supplementary Fig. S4E, F). Notably, several BAs varying from primary to secondary BAs did not show similar binding, suggesting the selective binding of DCA with METTL3 (Supplementary Fig. S5A–D). To further address this hypothesis, we performed co-immunoprecipitation experiments. As expectedly, METTL3 was found to form a complex with METTL14 and WTAP under no DCA treatment; on the contrary, METTL3 dissociation from METTL14 and WTAP was observed after DCA treatment (Fig. 6d). These results supported the hypothesis that DCA disrupted the METTL3–METTL14–WTAP complex by binding to METTL3, thus leading to a decrease of miR-92b-3p by affecting the process of [m^6^A] pri-miR-92b (Fig. 6e).

### DCA suppresses GBC tumor growth by downregulating miR-92b-3p expression

**Fig. 7:**
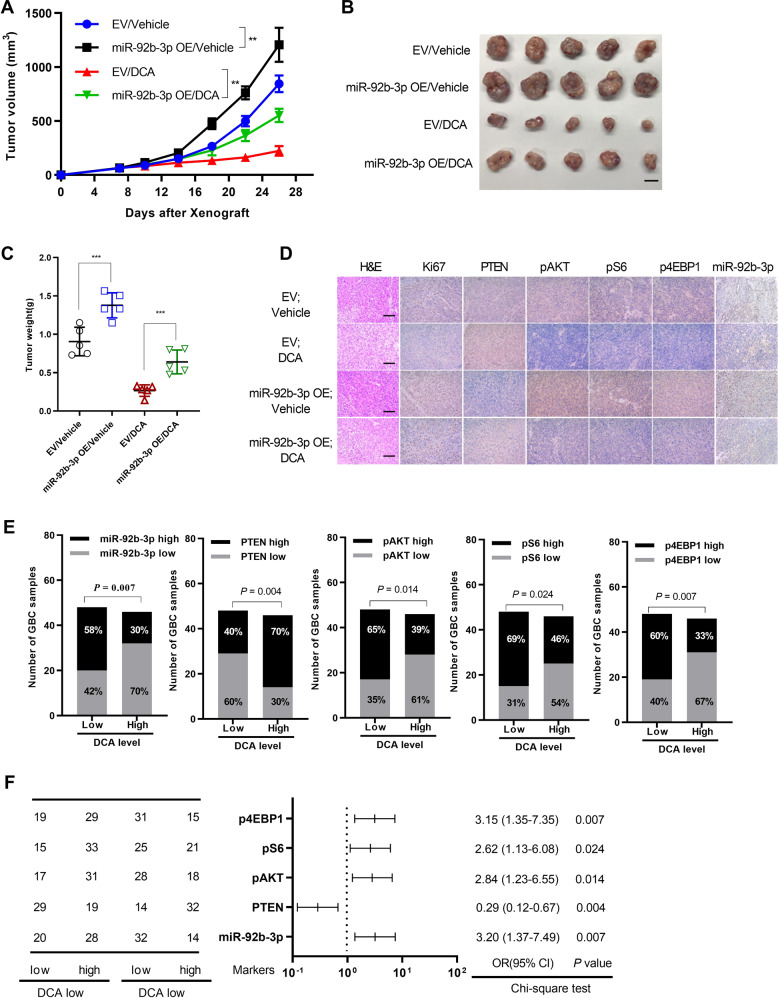
miR-92b-3p partly attenuates DCA-mediated abolishment in GBC progression. **a** Follow-up of tumor growth after nude mice were subcutaneously injected with NOZ cancer cells overexpressing miR-92b-3p (miR-92b-3p OE) or empty vector (EV), followed by feeding food with or without DCA. (*n* = 5 per group; two-way analysis of variance (ANOVA), comparisons at day 26 are shown; ***p < 0.001). **b** Excised xenograft tumors 4 weeks from **a**. Scale bars represent 10 mm. **c** Tumor weight in **b** are shown (**p < 0.01). **d** Representative images of Ki-67, PTEN, p-AKT (Ser473), p-70S6K (Thr389), p-eIF4EBP1 (phospho T37), and miR-92b-3p immunohistochemical (IHC) staining in **b** are shown. Scale bars represent 100 μm. Correlations between DCA serum level and miR-92b-3p RNA levels, PTEN, p-AKT (Ser473), p-70S6K (Thr389), and p-eIF4EBP1 (phospho T37) protein levels in GBC tissues (*n* = 94) (**e**) were evaluated by using Chi-square test (**f**).

To further explore the critical role of miR-92b-3p in GBC, we subcutaneously inoculated ectopically expressed miR-92b-3p or the empty vector as a control in NOZ cells into nude mice, which were then fed food containing DCA or equal amount of vehicle. Compared with the empty vector, overexpression of miR-92b-3p significantly increased the cell proliferation, whereas DCA treatment could significantly attenuated miR-92b-3p-mediated tumor growth (Fig. 7a). Consistently, the tumors excised from mice with miR-92b-3p-overexpressing cells increased weights compared with those excised from mice inoculated with the empty vector, even when treated with DCA (Fig. 7b, c). Similarly, immunohistochemical measurement of Ki-67 as well as p-AKT (Ser473), p-70S6K (Thr389), and p-eIF4EBP1 (phospho T37) showed remarkably lower proliferation rates coupled with significantly decreased levels of AKT signaling pathway after treatment with DCA; miR-92b-3p overexpression partially rescued the DCA-induced growth inhibition by targeting PTEN in GBC tumors (Fig. 7d).

Next, we assessed the correlation between DCA level in serum and miR-92b-3p level in GBC tissue samples. The results showed that the DCA level in serum was negatively correlated with miR-92b-3p level in GBC tissue samples (Fig. 7e; Supplementary Fig. S6). Given that miR-92b-3p targeted PTEN to inhibit AKT signaling pathway, we then measured PTEN, p-AKT (Ser473), p-70S6K (Thr389), and p-eIF4EBP1 (phospho T37) protein levels in GBC tissue samples. As expected, we found that all these proteins except for PTEN were remarkable negatively correlated with DCA level in serum, which was lower in GBC (Fig. 7e, f). Taken together, these results demonstrated that the DCA-miR-92b-3p-PTEN-PI3K/AKT regulatory axis existed in GBC tissues, which might provide a novel therapeutic strategy for GBC.

## Discussion

Our results showed that DCA levels in serum were downregulated in patients with GBC, which could contribute to tumor formation by increasing m^6^A modification level of pri-miR-92b, which targeted PTEN gene and promoted PI3K/AKT pathway signaling. More importantly, GBC patients with lower DCA levels showed a more shortened overall survival time. These findings revealed the important roles of DCA in GBC progression.

Recent evidence has shown that BAs affect a plethora of cellular processes including glucose, lipid, and energy metabolism, as well as immune response [9–11]. Although contradictory findings suggest that BAs exert both protective and cytotoxic effects on cells, further investigation revealed that the impact of BAs on cell fate is determined by cell types as well as BA subsets, in which conjugate BAs usually promote cell proliferation, whereas free BAs inhibit cell survival [17–20]. However, the role of BAs in GBC still remains obscure. In this study, we found that BAs homeostasis was abnormal in patients with GBC. Compared with that in normal individuals, DCA level in serum was low in patients with GA and GBC, and the level was lower in patients with GBC than that in patients with GA. Moreover, lower DCA level seemed to be correlated with poorer clinical outcome. Interestingly, DCA treatment significantly inhibited GBC cell proliferation and tumor growth of xenograft GBC models. In addition, our data showed that LCA and CDCA were also markedly changed in patience with GBC. Since that LCA and CDCA are implicated in the cancer [48–53], it is worth investigating the role of LCA and CDCA in gallbladder cancer. Furthermore, it is well-characterized that gut microbiota such as the genera Bacteroides, Eubacterium, and Clostridium are involved in process of secondary BAs via enzymatic modifications. Interestingly, the microbiome was recently identified in gallbladder [10]. Thus, microbiome alteration in gallbladder may affect secondary BAs metabolome. Further research involved in interaction between microbiome and secondary BAs metabolome in gallbladder is valuable.

Although miRNAs are known to play crucial roles in many cancers [43], their biological function in GBC remains poorly characterized and understood. We found that 26 miRNAs were significantly differentially expressed in DCA-treated GBC cells, in which miR-92b-3p was the most downregulated miRNA. MiR-92b-3p, a miRNAs derived from miR17-92 cluster located on chromosome 13, is involved in various human cancer as an oncogene [54–56]. Our data showed that it was upregulated in GBC tumor samples and the ectopic expression of miR-92b-3p highly enhanced GBC cell proliferation. Consistently, the xenograft mouse models of GBC cells also exhibited miR-92b-3p in pro-tumorigenic role. More importantly, miR-92b-3p partly ameliorated DCA-mediated suppression of cell proliferation and tumor growth. These data demonstrated that DCA could control the expression level of miR-92b-3p, an oncogenic factor, in GBC cells. Furthermore, we identified PTEN, a much known tumor suppressor [57, 58], as the direct target of miR-92b-3p. We found that downregulation of miR-92b-3p led to increased PTEN levels in the GBC cells. PTEN mainly functions in regulation of the PI3K/AKT signaling pathway [58], indeed, our data revealed that DCA increased PTEN by reducing miR-92b-3p level, and thus impaired the PI3K/AKT signaling in GBC cells.

Several studies have reported that RNA m^6^A methylation is closely involved in miRNAs maturation and serves as a crucial regulator in various cellular processes [45, 59]. Very excitingly, we observed that the m^6^A level of pri-miR-92b levels were significantly reduced in GBC cells under treatment with DCA. The underlying mechanism was revealed that DCA blocked the formation of the METTL3/METTL14/WTAP complex, which is responsible for m^6^A modification in diverse RNAs in mammalian [46, 47], by direct binding to METTL3 other than affecting their expressions. As expected, the m^6^A methylation of pri-miR-92b was impaired and sub-sequentially miR-92b-3p maturation was reduced when treated with DCA. It is of note that METTL3-mediated m^6^A methylation plays a role in promoting tumor progression in many cancers [46, 60, 61], thus it is worth exploring the role of DCA in other cancers. Since obeticholic acid, a synthetically modified BA, has been used to treat liver diseases, including noncirrhotic and nonalcoholic steatohepatitis, without obvious adverse effects [62]. Based on our data, we similarly propose that DCA might be used as an alternative therapeutic target in patients with GBC irrespective of whether they are chemotherapy resistant.

## Materials and methods

### Clinical materials

Surgically removed 243 GBC and paired non-tumor tissue samples were obtained from patients who underwent gallbladder-ectomy at Department of Biliary-Pancreatic Surgery, Renji Hospital affiliated to Shanghai Jiao Tong University School of Medicine between 2008 and 2018 with the patients’ consent. All the study protocols were approved by The Ethics Committees of Renji Hospital with written informed consent.

### Cell lines and cell culture

Human embryonic kidney 293T (HEK293T) cells and human GBC cell lines (NOZ, GBC-SD, and EGH1) were described in previous studies [5]. For the details of reagents, cell lines, and cell culture, see the Supplementary of “Materials and Methods”.

### Animal studies

For xenograft tumor model experiments, 2 × 10^6^ cells GBC-SD or NOZ cells were resuspended with 100 μl of 1 × PBS and subcutaneously into the flanks of BALB/c nude mice (male, 4–6-week old). Six days after injection, mice were randomly allocated to control and treatment groups. All mice were sacrificed and tumors were embedded in paraffin for tissue staining. In vivo studies were approved by the Institutional Animal Care and Use Committee of Renji Hospital affiliated to Shanghai Jiao Tong University School of Medicine.

### Plasmids and shRNAs

The open reading frame (ORF) sequences of METTL3, DROSHA, and DGCR8 were cloned into the pLenti-CDH-IRES-Puromycin vector (Addgene, USA) with the Flag-tag in the N-terminus, and the 3′-UTR sequence (+5908 to +6430) of PTEN at the downstream of its ORF was cloned into the pMir-Glo backbone (Promega). The full-length Human pre-miR-92b cDNA synthesized by Biosun (Shanghai, China) was subcloned into the pLenti-CMV-IRES-Puromycin vector (Addgene, USA). The wild-type and m6A-motification site mutant sequences of pri-miR-92b were synthesized by Biosun (Shanghai, China) and subcloned into the pLenti-CMV-IRES-Puromycin vector, respectively. All shRNAs used in this study were obtained from Department of Biochemistry and Molecular Cell Biology, Shanghai Jiao Tong University School of Medicine, except the construct for the depletion of miR-92b-3p which was generated by subcloning the sequence targeting hsa-miR-92b-3p synthesized by Biosun (Shanghai, China) into pLKO.1 (Addgene, USA). The sequences were listed in Supplementary Table S3.

### Transfection of oligos

All sequences for oligos and probes are included in Supplementary Table S3. The transfection reagent Lipofectamine 2000 and miRNAs mimics were purchased from ThermoFisher (USA). Transfection was followed manufacturer’s instructions. All experiments were performed 48 h after transfection.

### Lenti-pseudovirus production and transduction

Lenti-pseudovirus production and transduction were described in previous studies [5]. For the details, see the Supplementary of “Materials and Methods”.

### Cell proliferation assays

Cells were seeded in 96-well plates (2000 cells per well) treated with indicated concentration of DCA (50 μM) or vehicle and after a certain time of culturation, cell growth rate was measured using CCK-8 assays (Dojindo, Japan), followed by detection of the absorbance at 450 nm through Synergy 2 microplate reader (Biotek, USA). Each experiment with three replicates was repeated three times.

### Cytotoxic assays in vitro

Cells were seeded in 96-well plates (2000 cells per well) then treated with increasing concentrations of DCA for indicated times. Cell viability was assessed by the Cell Counting Kit-8 (Dojindo) assay. The absorbance at 450 nm was measured thorough a Synergy 2 microplate reader (Biotek). Each experiment with three replicates was repeated three times.

### Colony formation assay

Cells were seeded in 6-well plates (1000 cells per well) for 10 days until colonies were visible, and then treated with 50 μM DCA or vehicle for 48 h. The colonies were fixed with 4% paraformaldehyde and followed by staining with 0.1% crystal violet.

### Luciferase reporter assay

NOZ or GBC-SD cells grown in a 24-well plate were cotransfected with indicated miR-92b-3p mimics or equal amount of mock, and 50 ng of firefly luciferase reporter comprising wild-type or mutant 3′-UTR of PTEN fragment by using Lipofectamie 2000 (Invitrogen). Forty-eight hours after transfection, the luciferase assay was measured by the Dual-Luciferase Kit (Promega). Renilla luciferase activity was determined to normalize the firefly luciferase activity. Each experiment with three replicates was repeated three times.

### Quantitative real-time PCR

qRT-PCR were described in previous studies [5]. For the details, see the Supplementary of “Materials and Methods”. The primer sequences are shown in Supplementary Table S3.

### Immunoblotting

Immunoblotting were described in previous studies [5]. For the details, see the Supplementary of “Materials and Methods”.

### Co-immunoprecipitation (Co-IP)

Cells were lysed with RIPA buffer (25 mM Tris, pH 7.4, 150 mM NaCl, 5% Glycerol, 1% NP-40, 1 mM EDTA) supplemented with protease/phosphatase inhibitor cocktail (Pierce). Cell debris were removed by centrifugation and the supernatants were harvested then treated with RNase A (20 μg/ml) or RNase inhibitor (200 U/ml, New England Biolab) following immunoblotting or immunoprecipitation with antibodies indicated. The immunoblotting signals were detected by the ECL Kit (Millipore, Germany).

### Drug affinity responsive targets stability (DARTS)

DARTS was performed to determine the potential targets of DCA. Briefly, 50 × 10^6^ cells were lysed with M-PER (78501, Thermo Fisher Scientific) supplemented with protease/phosphatase inhibitor cocktail (Pierce). TNC buffer (50 mM Tris-HCL pH8.0, 50 mM NaCl, and 10 mM CaCl_2_) was added to the lysates. The supernatants were incubated with increasing concentration of DCA or EtOH (vehicle) for 1 h at room temperature following digestion with Pronase (1:3000) (Cat #10165921001, Roche) for 30 min. The digestion was terminated with protease inhibitor cocktail following ice incubation immediately. The immunoblotting signals were detected by the ECL Kit. The experiments were performed with three biological replicates.

### Cellular thermal shift assay (CETSA)

Cells were pretreated with 50 μM DCA for 24 h prior to subject to the CETSA protocol. Cells were washed with PBS containing protease inhibitor cocktail and transferred into PCR tubes. The cells were subjected to heat shocked with indicated temperature for 5 min to denature proteins, then were immediately cooled down at room temperature for 5 min. All the samples were next undergone three freeze-thaw cycles to lyse cells. The supernatant was subjected to immunoblotting and the band density was detected. The experiments were performed with three biological replicates.

### RNA sequencing and data analysis

Total RNA was extracted with TRIzol reagent according to the manufacturer’s instructions, followed by preparing RNA-seq libraries with NEB Next_Ultra RNA Library Prep Kit. Then the libraries were subjected to deep sequence with Illumina Hi-Seq platform. The reads were mapped to NCBI human genome GRCh38 by STAR. The gene expression levels of each transcript were calculated as RPKM (the number of reads per kilobase of exon model per million mapped reads). Differential gene expression was estimated by R Bioconductor DESeq2 package.

### miRNA expression analysis (NGS)

For miRNAs-seq, total RNA of two pairs NOZ cells treated with vehicle or DCA were extracted using the miRNeasy Micro Kit (Qiagen). RNA-seq libraries were obtained by using the NEBnext Multiplex Small RNA Library Prep Set (New England Biolabs), following the manufacturer’s instructions. Sequencing was performed on Illumina Hi-Seq4000 platform with 50 bp single end reads using Illumina reagents according to the manufacturer’s instructions.

### Analysis of miRNA target genes in silico

The miRNA target genes were predicted using publicly available algorithms: DIANA (http://diana.imis.athena-innovation.gr/DianaTools/index.php?r=microT_CDS/index), miRNAorg (http://www.microrna.org/microrna/getGeneForm.do), and TargetScan (http://www.targetscan.org/vert_71/) in silico. Then the target genes overlapping from each database were ranged by confidence scores. The target genes with highest confidence score were then selected to further analysis.

### MiRNA in situ hybridization and immunohistochemistry

The sequence of the human miR-92b-3p probe, U6 probe (positive control), and scramble probe (negative control) were shown in Supplementary Table S3. To quench endogenous peroxidase activity, paraffin tissue slides were firstly deparaffined, and then rehydrated. The slides next were incubated in miRNA Hybridization solution, followed by incubation with miR-92b-3p probe. The samples were then washed, followed by staining with 3, 3′-Diaminobenzidine. Immunohistochemistry assays were conducted as reported previously [5].

### Northern blot assays

Total RNA were electrophoresed through formaldehyde gel electrophoresis and then transferred to a Biodyne Nylon membrane (Millipore). The membrane was then pretreated with hybridization buffer (DIG Easy Hyb Granules, Roche) at 37 °C for 30 min in a hybridization oven, followed by hybridization with buffer containing the indicated denatured probes at 65 °C for 12 h. After washing, the membrane was incubated with anti-Digoxigenin-AP antibody (Roche) at room temperature for 30 min. Finally, the membrane was developed with CDP-Star (Roche) for 5 min and exposed to X-ray films.

### RNA immunoprecipitation assay (RIP)

Total RNA was extracted with TRIzol reagent according to the manufacturer’s instructions. Then preincubation of total RNA (input control), isotype control (IgG), or AGO2 antibody with protein A beads in RIP buffer (150 mM NaCl, 10 mM Tris-HCL and 0.1% NP-40) were conducted, followed with qRT-PCR to estimate the co-precipitated RNAs.

### MeRIP-qPCR

Total RNA were subjected to fragment, then incubated with protein A beads previously bound anti-m6A polyclonal antibody (Synaptic Systems) or IgG (isotype control) in RIP buffer at 4 °C for 3 h. Next, samples were washed, followed by purification with Qiagen and subjected to qRT-PCR.

### In vitro pri-miRNA processing assays

For the details, see the Supplementary of “Materials and Methods”. Briefly, the pri-miR-92b was transcript in vitro. To obtain [m6A] pri-miR-92b, ATP in the transcription reaction was instead by N6-methyl-ATP (m6A). In addition, the mutant pri-miR-92b, with A to T mutation at the m^6^A site, was subjected to in vitro transcription, and followed by in vitro processing.

### UHPLC-MS/MS

Detection of serum BAs was performed by UHPLC-MS/MS system. Total bloods of human samples were collected. Then, serum was isolated into new tubes and centrifuged. Upon centrifugation, the supernatant was subjected to UHPLC-MS/MS measurement. The BAs and their conjugates were analyzed by a multiple reaction monitoring mode.

### Statistical analysis

The data were presented as the mean ± SEM or SD, and represent at least three independent experiments. To estimate the effects of variables on survival, we utilized the univariate and multivariate Cox proportional hazards model. For the analysis of progression-free survival and overall survival, the Kaplan–Meier method test was used. All statistical analyses were performed by SPSS 20.0 software and a *p* value < 0.05 was determined to statistical significance.

## Supplementary information

Supplementary Information
