# Supplementary material for: Deoxycholic acid modulates the progression of gallbladder cancer through N6-methyladenosine-dependent microRNA maturation
Source: Oncogene. 2020 Jun 8;39(26):4983–5000. doi: 10.1038/s41388-020-1349-6 (PMC7314665; doi:10.1038/s41388-020-1349-6)
Supplement: Supplementary file 1 — Supplementary Information [file 41388_2020_1349_MOESM1_ESM.pdf]

Supplementary Information for

Deoxycholic acid modulates the progression of gallbladder cancer through N6-methyladenosine-dependent microRNA maturation

Supplementary Figures

Supplementary Figure. S1 Related to Figure 1.

Supplementary Figure. S2 Related to Figure 3.

Supplementary Figure. S3 Related to Figure 4.

Supplementary Figure. S4 Related to Figure 5-6.

Supplementary Figure. S5 Related to Figure 6.

Supplementary Figure. S6 Related to Figure 7.

Supplementary Tables

Supplementary Table S1 Relationship between DCA levels and clinicpathologic features of GBC.

Supplementary Table S2 Relationship between miR-92b-3p levels and clinicpathologic features of GBC.

Supplementary Table S3 Sequence of oligonucleotide used in this study.

Materials and Methods

Figure. S1

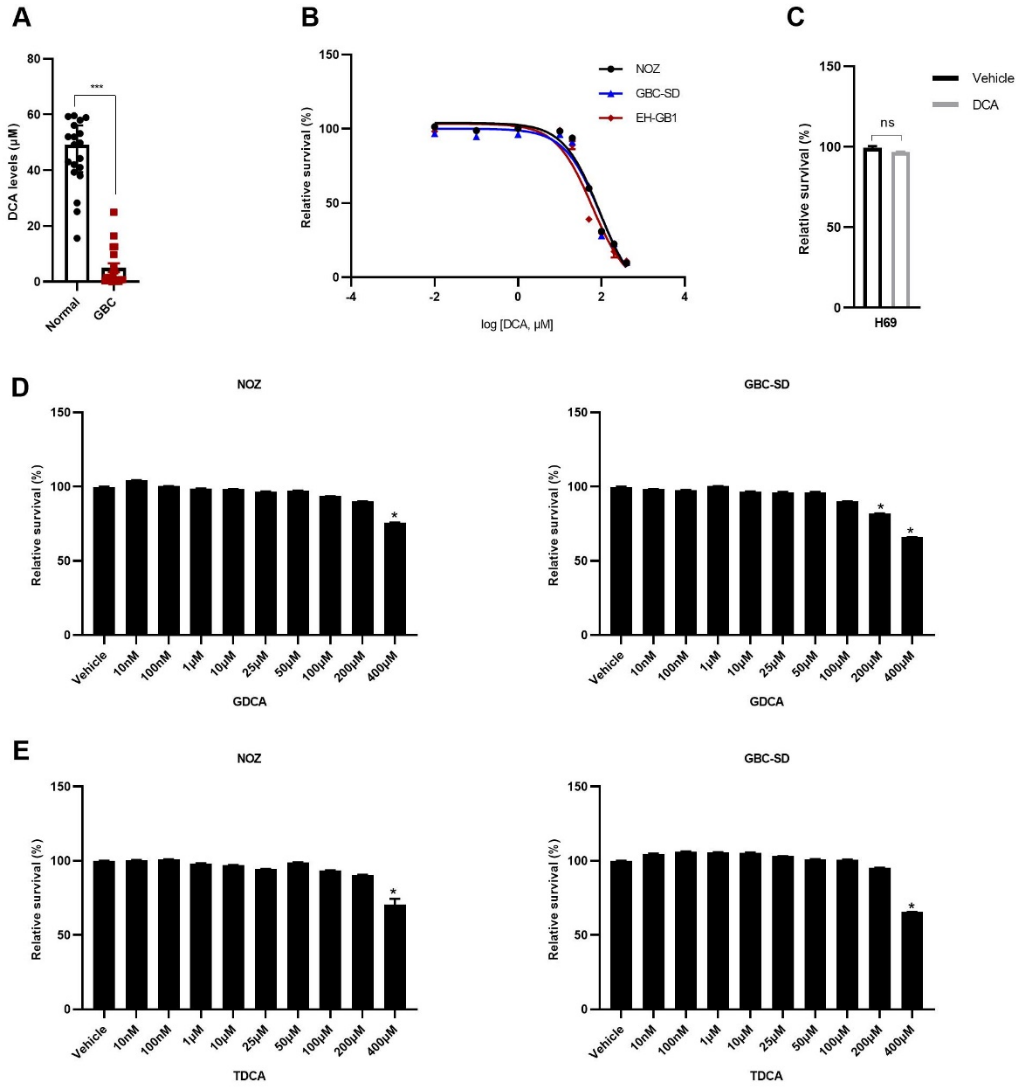
A
B
C
DCA levels (μM)
***
Normal
GBC
Relative survival (%)
log [DCA, μM]
NOZ
GBC-SD
EH-GB1
ns
Vehicle
DCA
H69
D
NOZ
GBC-SD
Vehicle
10nM
100nM
1μM
10μM
25μM
50μM
100μM
200μM
400μM
GDCA
E
TDCA

Supplementary Figure. S1 Related to Figure 1.

A, Levels of DCA in gallbladder from normal individuals and GBC patients (means ± SEM, n = 18 vs. 18; ****P* < 0.001). **B,** Relative survival of NOZ, GBC-SD and EH-GB1 cells treated with increasing concentrations of DCA. **C,** H69, a well-established non-malignant cholangiocytes (originate from bile duct epithelium), could survive well treated with DCA. **D,** The higher dosage of GDCA impair the cell viability of NOZ and GBC-SD. One-way analysis of variance (ANOVA). (means ± SEM, **P* < 0.05). **E,** The higher dosage of TDCA impair the cell viability of NOZ and GBC-SD. One-way analysis of variance (ANOVA). (means ± SEM, **P* < 0.05).

Figure. S2

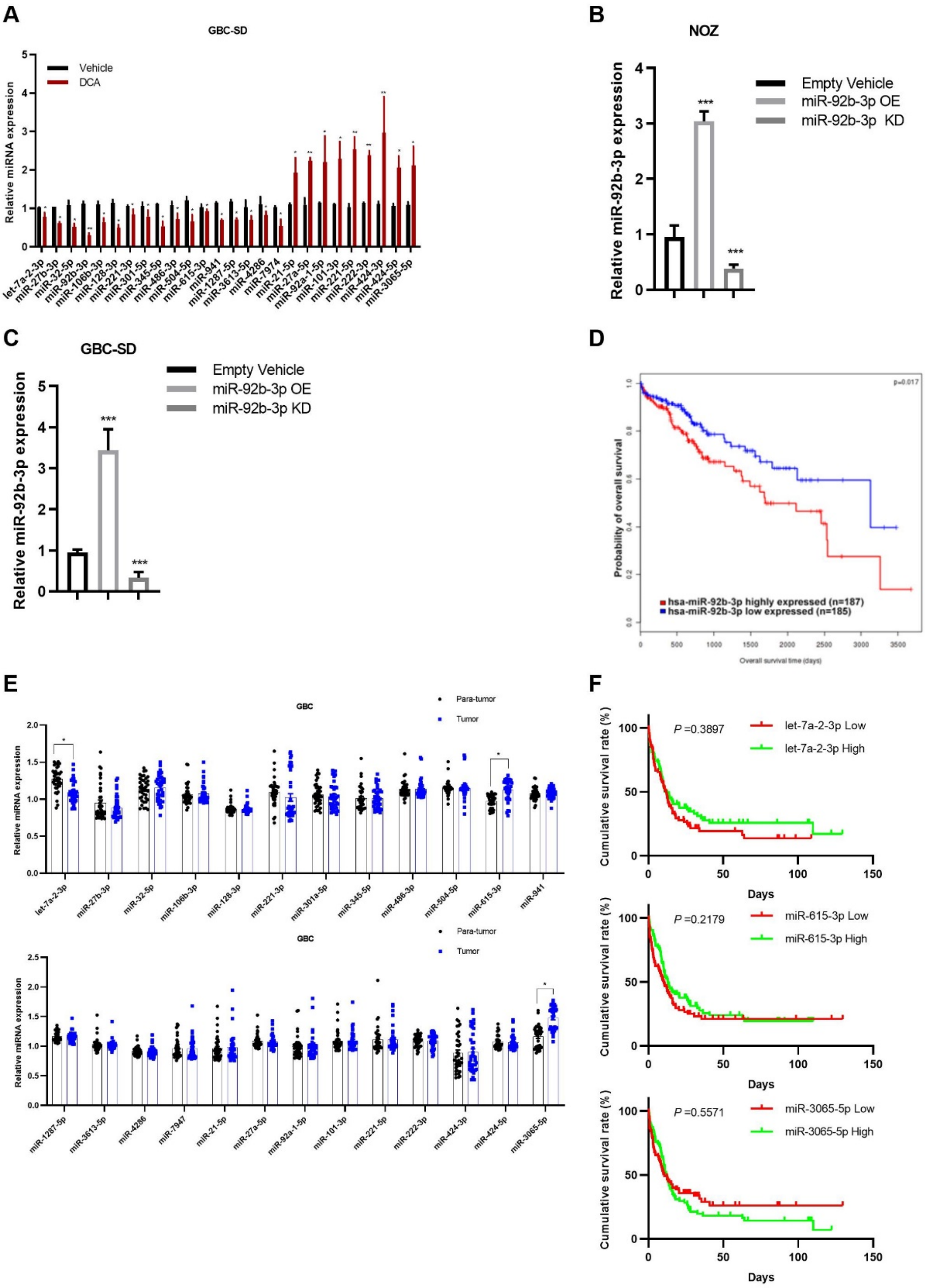

A
GBC-SD
Vehicle
DCA
Relative miRNA expression
let-7a-2-3p
miR-27b-3p
miR-32-5p
miR-92b-3p
miR-106b-3p
miR-128-3p
miR-221-3p
miR-301-5p
miR-345-5p
miR-486-3p
miR-504-5p
miR-615-3p
miR-941
miR-1287-5p
miR-3613-5p
miR-4286
miR-7974
miR-21-5p
miR-27a-5p
miR-92a-1-5p
miR-101-3p
miR-221-5p
miR-222-3p
miR-424-3p
miR-424-5p
miR-3065-5p
B
NOZ
Empty Vehicle
miR-92b-3p OE
miR-92b-3p KD
Relative miR-92b-3p expression
***
C
GBC-SD
Empty Vehicle
miR-92b-3p OE
miR-92b-3p KD
Relative miR-92b-3p expression
***
D
p=0.017
Probability of overall survival
hsa-miR-92b-3p highly expressed (n=187)
hsa-miR-92b-3p low expressed (n=185)
Overall survival time (days)
E
GBC
Para-tumor
Tumor
Relative miRNA expression
let-7a-2-3p
miR-27b-3p
miR-32-5p
miR-106b-3p
miR-128-3p
miR-221-3p
miR-301a-5p
miR-345-5p
miR-486-3p
miR-504-5p
miR-615-3p
miR-941
GBC
Para-tumor
Tumor
Relative miRNA expression
miR-1287-5p
miR-3613-5p
miR-4286
miR-7947
miR-21-5p
miR-27a-5p
miR-92a-1-5p
miR-101-3p
miR-221-5p
miR-222-3p
miR-424-3p
miR-424-5p
miR-3065-5p
F
Cumulative survival rate (%)
P =0.3897
let-7a-2-3p Low
let-7a-2-3p High
Days
Cumulative survival rate (%)
P =0.2179
miR-615-3p Low
miR-615-3p High
Days
Cumulative survival rate (%)
P =0.5571
miR-3065-5p Low
miR-3065-5p High
Days

Supplementary Figure. S2 Related to Figure 3.

A, Expression levels of indicated miRNAs in GBC-SD cells treated with DCA or vehicle. Results represent means ± standard derivation (S.D.) from three independent measurements. **B-C,** RNA level of miR-92b-3p in NOZ (**B**) and GBC-SD (**C**) cells overexpressing or knockdown of miR-92b-3p. Data shown are means ± S.D. from three representative independent experiments. **D,** Kaplan–Meier survival curves of overall survival (OS) according to miR-92b-3p level in liver hepatocellular carcinoma (LIHC) by using the online bioinformatics tool Kaplan–Meier Plotter (*$P < 0.05$). **E,** Expression levels of indicated miRNAs in 38 pairs of GBC tissues and adjacent normal tissues by using qPCR (two-way analysis of variance (ANOVA); *$P < 0.05$). **F,** Kaplan–Meier survival curves of overall survival according to let-7a-2-3p, miR-615-3p and miR-3065-5p level respectively.

Figure. S3

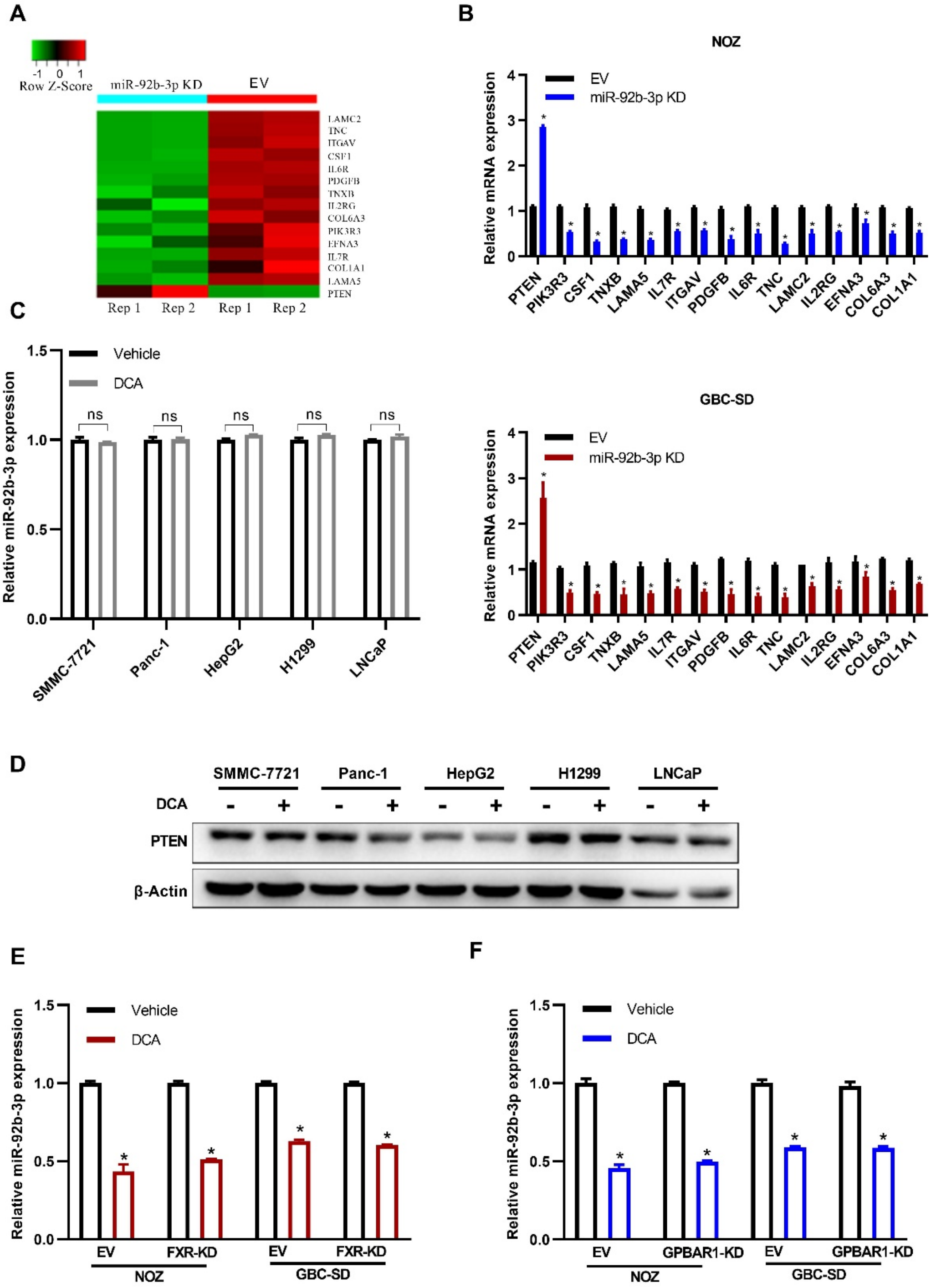
A
Row Z-Score
miR-92b-3p KD
EV
LAMC2
TNC
ITGAV
CSF1
IL6R
PDGFB
TNXB
IL2RG
COL6A3
PIK3R3
EFNA3
IL7R
COL1A1
LAMA5
PTEN
Rep 1
Rep 2
Rep 1
Rep 2
B
NOZ
GBC-SD
EV
miR-92b-3p KD
Relative mRNA expression
PTEN
PIK3R3
CSF1
TNXB
LAMA5
IL7R
ITGAV
PDGFB
IL6R
TNC
LAMC2
IL2RG
EFNA3
COL6A3
COL1A1
C
Vehicle
DCA
ns
Relative miR-92b-3p expression
SMMC-7721
Panc-1
HepG2
H1299
LNCaP
D
SMMC-7721
Panc-1
HepG2
H1299
LNCaP
DCA
PTEN
β-Actin
E
Vehicle
DCA
Relative miR-92b-3p expression
EV
FXR-KD
NOZ
GBC-SD
F
Vehicle
DCA
Relative miR-92b-3p expression
EV
GPBAR1-KD
NOZ
GBC-SD

Supplementary Figure. S3 Related to Figure 4.

A, Heatmap representation of significantly differentially expressed mRNAs associated with PI3K/AKT signalling ($P < 0.05$; fold change, >2 or <0.5) in NOZ cells with miR-92b-3p knockdown or empty vector. **B,** Expression levels of indicated mRNAs in NOZ (upper) and GBC-SD (down) cells as in a. Results represent means ± standard derivation (S.D.) from three independent measurements. **C,** The expression of miR-92b-3p in SMMC-7721, Panc-1, HepG2, H1299 and LNCaP treated with DCA. Data represent mean ± S.D. from three independent experiments. **D,** The expression of PTEN in SMMC-7721, Panc-1, HepG2, H1299 and LNCaP treated with DCA. **E,** The expression of miR-92b-3p in GBC cancer cells with or without FXR knockdown treated with DCA. Data represent mean ± S.D. from three independent experiments. **F,** The expression of miR-92b-3p in GBC cancer cells with or without GPBAR1 knockdown treated with DCA. Data represent mean ± S.D. from three independent experiments.

Figure. S4

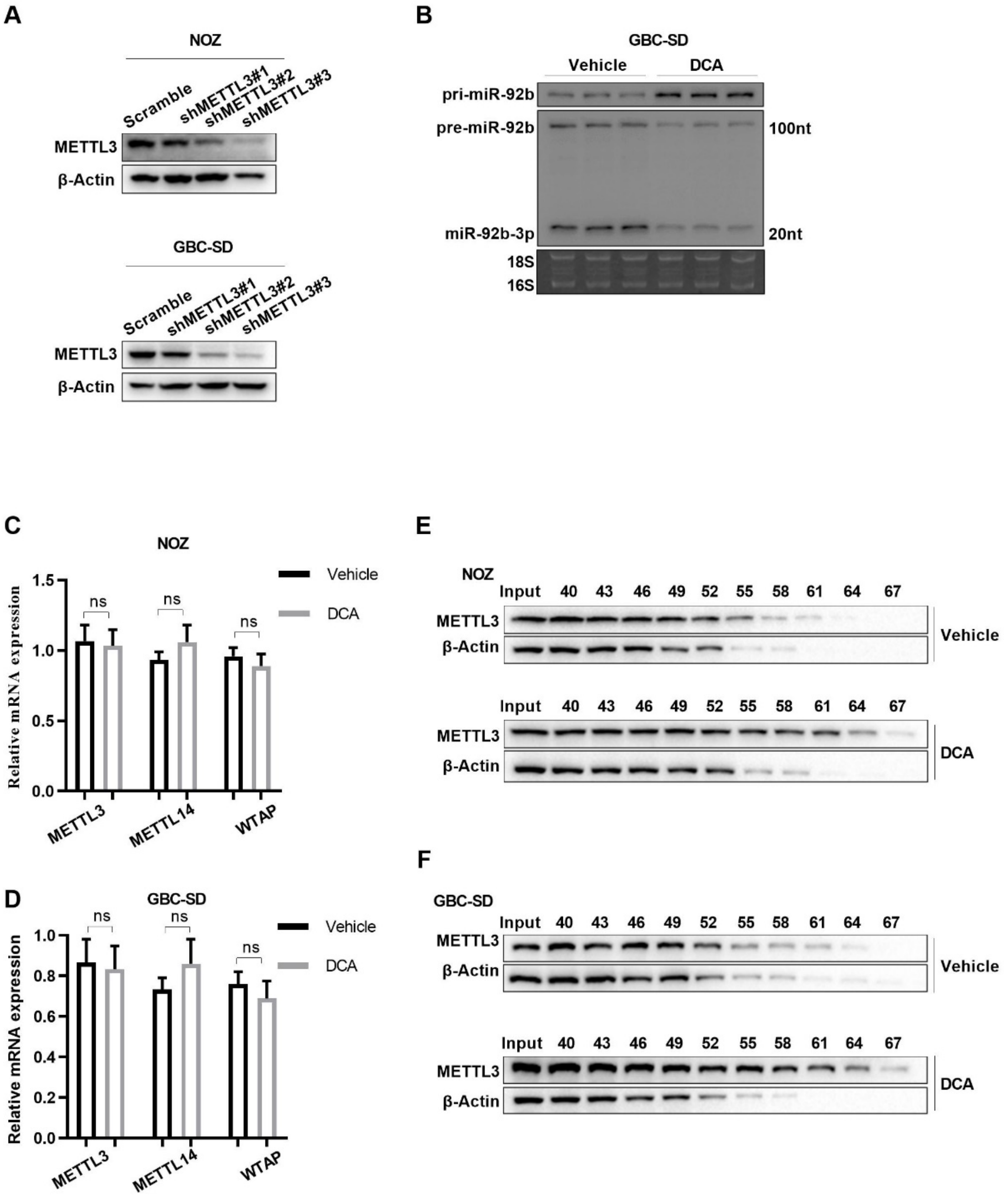
A
NOZ
Scramble
shMETTL3#1
shMETTL3#2
shMETTL3#3
METTL3
β-Actin
GBC-SD
Scramble
shMETTL3#1
shMETTL3#2
shMETTL3#3
METTL3
β-Actin
B
GBC-SD
Vehicle
DCA
pri-miR-92b
pre-miR-92b
100nt
miR-92b-3p
20nt
18S
16S
C
NOZ
Vehicle
DCA
Relative mRNA expression
ns
METTL3
METTL14
WTAP
D
GBC-SD
Relative mRNA expression
E
NOZ
Input 40 43 46 49 52 55 58 61 64 67
METTL3
β-Actin
Vehicle
DCA
F
GBC-SD

Supplementary Figure. S4 Related to Figure 5-6.

A, METTL3 protein levels in NOZ (upper) and GBC-SD (down) cells with or without METLL3 knockdown. **B,** Northern blot detection of the levels of pri-miR-92b, pre-miR-92b, and miR-92b-3p in GBC-SD challenged with DCA or vehicle. 28S and 18S rRNAs were used as loading controls. **C-D,** RNA levels of METTL3, METTL14 and WTAP in NOZ (**C**) and GBC-SD (**D**) cells treated with or without DCA. Data shown are means ± S.D. from three representative independent experiments. **E-F,** Representative western blots for the impact of DCA on thermal stabilization of METTL3 protein in NOZ (**E**) and GBC-SD (**F**) cells via CETSAs. The results are derived from three independent measurements.

Figure. S5

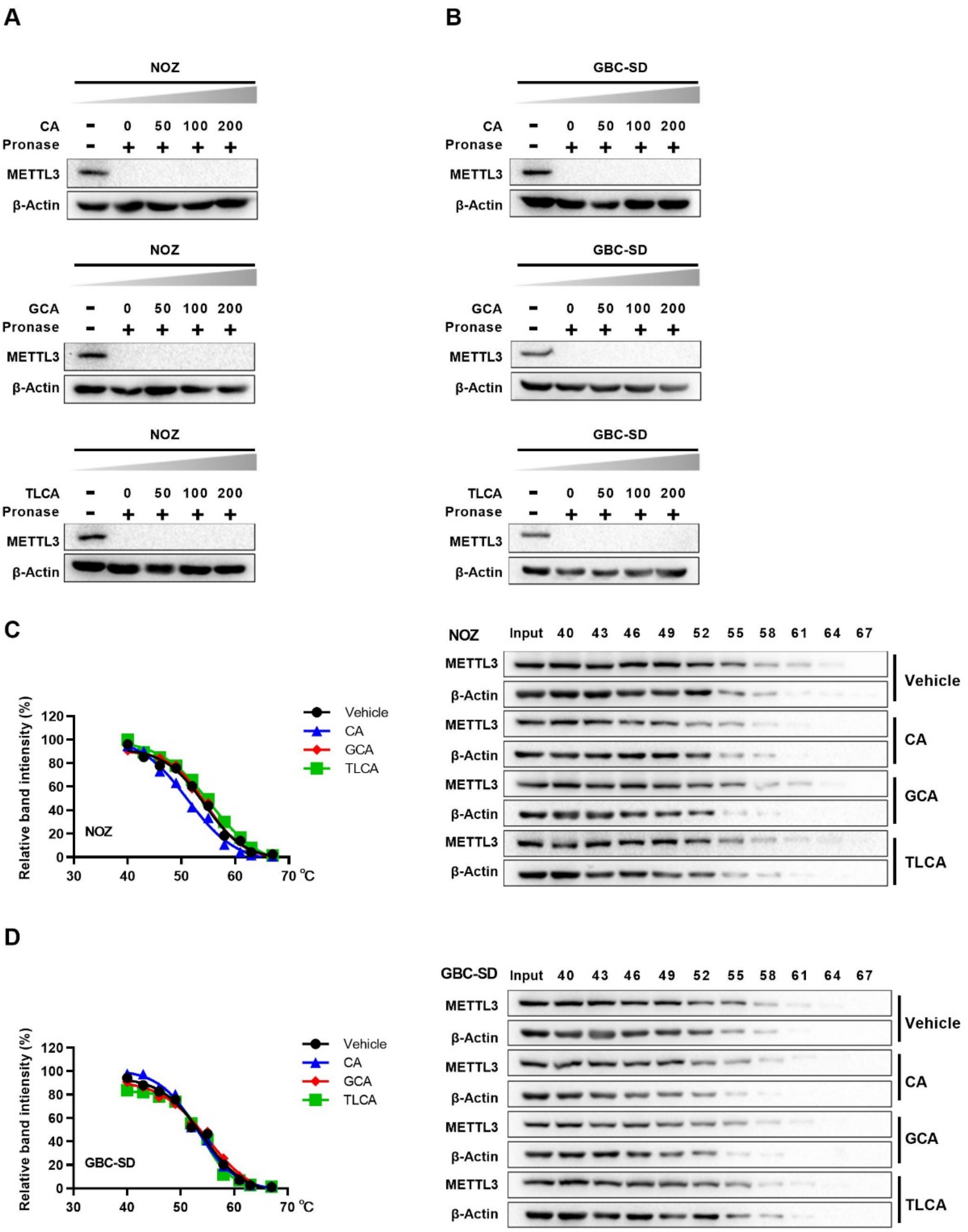
A
B
NOZ
GBC-SD
CA
GCA
TLCA
Pronase
0 50 100 200
METTL3
β-Actin
C
D
Relative band intiensity (%)
Vehicle
CA
GCA
TLCA
120
100
80
60
40
20
0
30 40 50 60 70 °C
Input 40 43 46 49 52 55 58 61 64 67

Supplementary Figure. S5 Related to Figure 6.

A-B, Measurement of the direct interaction of CA, GCA, TLCA with METTL3 in DARTS assays.

C-D, Identification of the binding affinity of CA, GCA, TLCA and METTL3 through CETSAs.

Figure. S6

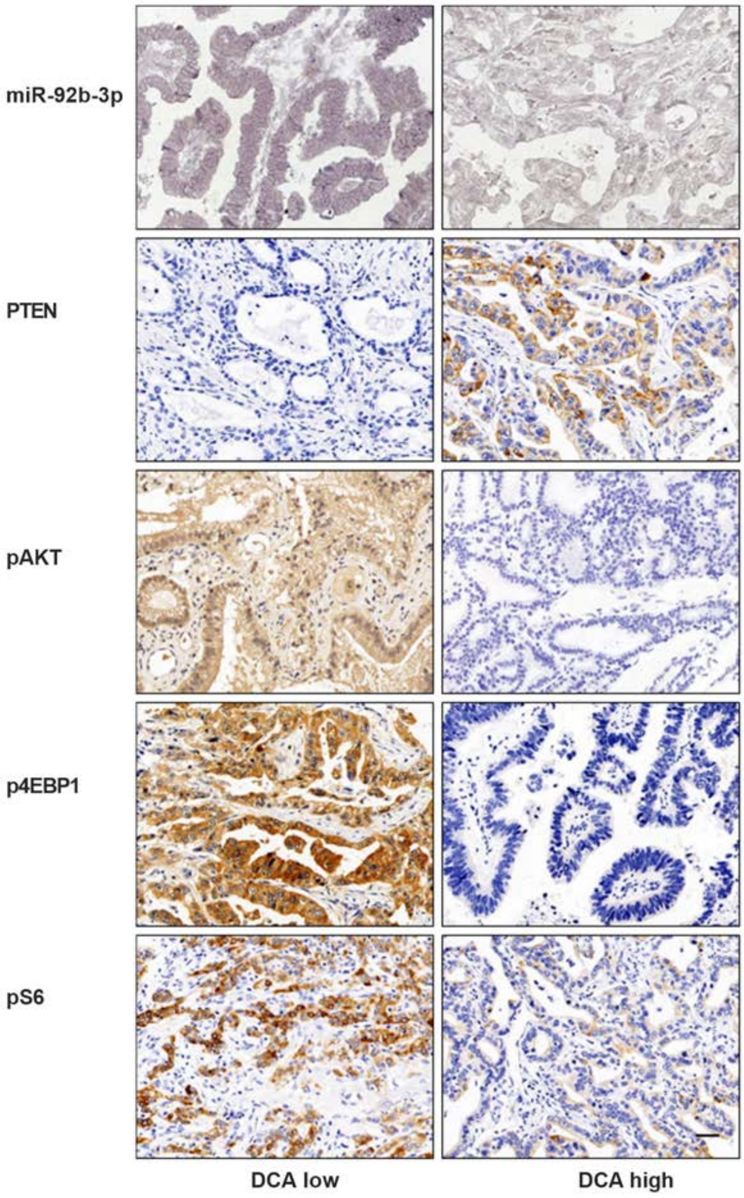

Supplementary Figure. S6 Related to Figure 7.

Representative images of immunohistochemical (IHC) staining in tumor sample from GBC patients with low or high DCA serum level (Figure. 7E-F) are shown. Scale bars represent 100 μm.

Supplementary Table 1. Relationship between DCA levels and clinicpathologic features of GBC patients.

Variable	All GBC patients (n, %)	DCA levels		[b]*P* value
		Low (n, %)	High (n, %)	
Age (continuous)				
Median (ranges)	64.9(38-86)	64.7(38-86)	65.0(41-84)	0.87
Follow-up time				
Median (ranges)	10.3(1.1-36.0)	7.9(1.5-32.8)	16.0(1.1-36.0)	2.6×10^{-3}
Age				
≤65 y	45(47.9)	19(39.6)	26(56.5)	0.10
>65 y	49(52.1)	29(60.4)	20(43.5)	
Gender				
Female	59(62.8)	28(58.3)	31(67.4)	0.36
Male	35(37.2)	20(41.7)	15(32.6)	
T stage (n, %)				
T1-T2	19(20.2)	6(12.5)	13(28.3)	0.06
T3-T4	75(79.8)	42(87.5)	33(71.7)	
Lymph-node metastasis				
No	53(56.4)	24(50)	29(63)	0.20
Yes	41(43.6)	24(50)	17(37)	
Distant metastasis				
No	79(84)	40(83.3)	39(84.8)	0.85
Yes	15(16)	8(16.7)	7(15.2)	
[a]TNM stage				
I-II	18(19.1)	5(10.4)	13(28.3)	0.03
III-IV	76(80.9)	43(89.6)	33(71.7)	
Tumor site				
Bottom/body	69(73.4)	32(66.7)	37(80.4)	0.13
Duct/neck	25(26.6)	16(33.3)	9(19.6)	
Tumor size				
≤3 cm	43(45.7)	15(31.3)	28(60.9)	4.0×10^{-3}
>3 cm	51(54.3)	33(68.8)	18(39.1)	
Liver invasion				
No	56(59.6)	27(56.3)	29(63)	0.50
Yes	38(40.4)	21(43.8)	17(37)	

[a]Tumor stage was defined according to the American Joint Committee on Cancer (AJCC) TNM staging system (AJCC 8th edition).

[b]Compared by student t test or by Pearson's χ^2 test.

Supplementary Table 2. Relationship between miR-92b-3p levels and clinicpathologic features of GBC patients.

Variable	All GBC patients (n, %)	miR-92b-3p levels		[b]*P* value
		Low (n, %)	High (n, %)	
Age (continuous)				
Median (ranges)	68(33-91)	68(42-91)	67(33-87)	0.9691
Follow-up time				
Median (ranges)	12.0(7.15-16.2)	16.2(1.2-129.6)	3.9(0.2-90.8)	$< 1\times10^{-3}$
Age				
≤65 y	70(47.0)	38(50.7)	32(43.2)	0.38
>65 y	79(53.0)	37(49.3)	42(56.7)	
T stage (n, %)				
T1-T2	67(45.0)	39(52.0)	28(37.8)	0.08
T3-T4	82(55.0)	36(48.0)	46(62.2)	
Lymph-node metastasis				
No	101(69.8)	54(72.0)	47(63.5)	0.22
Yes	48(30.2)	21(28.0)	27(36.5)	
Distant metastasis				
No	91(61.1)	54(59.3)	37(40.1)	0.15
Yes	58(38.9)	21(36.2)	37(63.8)	
[a]TNM stage				
I-II	67(45.0)	40(53.3)	27(36.5)	0.04
III-IV	82(55.0)	35(46.7)	47(63.5)	

[a]Tumor stage was defined according to the American Joint Committee on Cancer (AJCC) TNM staging system (AJCC 8th edition).

[b]Compared by student t test or by Pearson's χ^2 test.

Supplementary Table 3. Sequence of primers, shRNAs, miRNAs mimics or probe used in this study.

qPCR primers for mRNAs		
Gene	Forward	Reverse
METTL3	AAGCTGCACTTCAGACGAAT	GGAATCACCTCCGACACTC
METTL14	AGAAACTTGCAGGGCTTCCT	TCTTCTTCATATGGCAAATTTTCTT
WTAP	GGCGAAGTGTCGAATGCT	CCAACTGCTGGCGTGTCT
PTEN	CGTTACCTGTGTGTGGTGATA	CTCTGGTCCTGGTATGAAGAATG
PIK3R3	AGCAGACATCCTTGGTTCAG	CTCTCTTCCCACTTCCTCTTTATC
CSF1	CACCATGCGCTTCAGAGATAA	GGCCTTGTCATGCTCTTCATA
TNXB	GGTATTCACCCACCGCATTA	CCAACTCCTCCAGGATCTCTA
LAMA5	GCCTACGTCCTCATCAAGTTT	TTGGAGGAGGCAAAGAACTG
IL7R	CCTCTGCACCAGCAGTAATAA	ATCAAGGAGGTGGAAGGAATG
ITGAV	ACTCTTAGCTGGTCTTCGTTTC	TGTGAGATACAACTGGGCTTAC
PDGFB	GGTGGGTTAGAGATGGAGTTTG	GAACCAGAGGAAGAGGTGAATC
IL6R	CTCCCATCCCACCACATAAA	GGCACAGAATCACCCTTACT
TNC	CAAGGTGGGAGGATAGCTTAAC	CACTACCACGCTTGCCTAAT
LAMC2	CAACTGAGGTTCTTGGGATACA	GTTGACCTGAGCATACCCATTA
IL2RG	GTTCCTGACACAGACAGACTAC	GGCAGCTGCAGGAATAAGA
EFNA3	CTGGAAGTGTCTGAGGATGAAG	GCACGTTGATCTTCACATTGG
COL6A3	GCAGTCTGAGGACTCCTATTTG	CTGCTCAAGCTCTGCCTTAT
COL1A1	CTAAAGGCGAACCTGGTGAT	TCCAGGAGCACCAACATTAC
β-Actin	CCAACCGCGAGAAGATGA	CCAGAGGCGTACAGGGATAG

Stem-loop qPCR primers	
hsa-miR-let-7a-2-3p	GTCGTATCCAGTGCGTGTCGTGGAGTCGGCAATTGCACTGGATACGACGGAAAG
hsa-miR-27b-3p	GTCGTATCCAGTGCGTGTCGTGGAGTCGGCAATTGCACTGGATACGACGCAGAA
hsa-miR-32-5p	GTCGTATCCAGTGCGTGTCGTGGAGTCGGCAATTGCACTGGATACGACTGCAAC
hsa-miR-92b-3p	GTCGTATCCAGTGCGTGTCGTGGAGTCGGCAATTGCACTGGATACGACGGAGGC
hsa-miR-106b-3p	GTCGTATCCAGTGCGTGTCGTGGAGTCGGCAATTGCACTGGATACGACGCAGCA
hsa-miR-128-3p	GTCGTATCCAGTGCGTGTCGTGGAGTCGGCAATTGCACTGGATACGACAAAGAG
hsa-miR-221-3p	GTCGTATCCAGTGCGTGTCGTGGAGTCGGCAATTGCACTGGATACGACGAAACC
hsa-miR-301a-5p	GTCGTATCCAGTGCGTGTCGTGGAGTCGGCAATTGCACTGGATACGACAGTAGT
hsa-miR-345-5p	GTCGTATCCAGTGCGTGTCGTGGAGTCGGCAATTGCACTGGATACGACGAGCCC
hsa-miR-486-3p	GTCGTATCCAGTGCGTGTCGTGGAGTCGGCAATTGCACTGGATAC

	GACATCCTG
hsa-miR-504-5p	GTCGTATCCAGTGCGTGTCGTGGAGTCGGCAATTGCACTGGATAC GACGATAGA
hsa-miR-615-3p	GTCGTATCCAGTGCGTGTCGTGGAGTCGGCAATTGCACTGGATAC GACAAGAGG
hsa-miR-941	GTCGTATCCAGTGCGTGTCGTGGAGTCGGCAATTGCACTGGATAC GACGCACAT
hsa-miR-1287-5p	GTCGTATCCAGTGCGTGTCGTGGAGTCGGCAATTGCACTGGATAC GACGACTCG
hsa-miR-3613-5p	GTCGTATCCAGTGCGTGTCGTGGAGTCGGCAATTGCACTGGATAC GACGAACAA
hsa-miR-4286	GTCGTATCCAGTGCGTGTCGTGGAGTCGGCAATTGCACTGGATAC GACGGTACC
hsa-miR-7974	GTCGTATCCAGTGCGTGTCGTGGAGTCGGCAATTGCACTGGATAC GACGGGCTC
hsa-miR-21-5p	GTCGTATCCAGTGCGTGTCGTGGAGTCGGCAATTGCACTGGATAC GACTCAACA
hsa-miR-27a-5p	GTCGTATCCAGTGCGTGTCGTGGAGTCGGCAATTGCACTGGATAC GACTGCTCA
hsa-miR-92a-1-5p	GTCGTATCCAGTGCGTGTCGTGGAGTCGGCAATTGCACTGGATAC GACAGCATT
hsa-miR-101-3p	GTCGTATCCAGTGCGTGTCGTGGAGTCGGCAATTGCACTGGATAC GACTTCAGT
hsa-miR-221-5p	GTCGTATCCAGTGCGTGTCGTGGAGTCGGCAATTGCACTGGATAC GACAAATCT
hsa-miR-222-3p	GTCGTATCCAGTGCGTGTCGTGGAGTCGGCAATTGCACTGGATAC GACACCCAG
hsa-miR-424-3p	GTCGTATCCAGTGCGTGTCGTGGAGTCGGCAATTGCACTGGATAC GACATAGCA
hsa-miR-424-5p	GTCGTATCCAGTGCGTGTCGTGGAGTCGGCAATTGCACTGGATAC GACTTCAAA
hsa-miR-3065-5p	GTCGTATCCAGTGCGTGTCGTGGAGTCGGCAATTGCACTGGATAC GACTCCAGC

qPCR primers for miRNAs		
hsa-miR-let-7a-2-3p	CGCGCACTGTACAGCCTCCTAG	CAGTGCGTGTCGTGGAGTC
hsa-miR-27b-3p	CGCGCATTCACAGTGGCTAAG	CAGTGCGTGTCGTGGAGTC
hsa-miR-32-5p	CGCACGCATATTGCACATTACTAA	CAGTGCGTGTCGTGGAGTC
hsa-miR-92b-3p	CGCATATTGCACTCGTCCCG	CAGTGCGTGTCGTGGAGTC
hsa-miR-106b-3p	CACCGCACTGTGGGTACT	CAGTGCGTGTCGTGGAGTC
hsa-miR-128-3p	ACGCATCACAGTGAACCGGT	CAGTGCGTGTCGTGGAGTC
hsa-miR-221-3p	CACGCAAGCTACATTGTCTGCTG	CAGTGCGTGTCGTGGAGTC
hsa-miR-301a-5p	CACGCAGCTCTGACTTTATTGC	CAGTGCGTGTCGTGGAGTC
hsa-miR-345-5p	CACGCAGCTGACTCCTAGTCCA	CAGTGCGTGTCGTGGAGTC

hsa-miR-486-3p	CGCACGGGGCAGCTCAGTA	CAGTGCGTGTCGTGGAGTC
hsa-miR-504-5p	CAAGACCCTGGTCTGCACTCTATC	CAGTGCGTGTCGTGGAGTC
hsa-miR-615-3p	CATCCGAGCCTGGGTCTC	CAGTGCGTGTCGTGGAGTC
hsa-miR-941	CACCCGGCTGTGTGCAC	CAGTGCGTGTCGTGGAGTC
hsa-miR-1287-5p	CGCATGCTGGATCAGTGGTT	CAGTGCGTGTCGTGGAGTC
hsa-miR-3613-5p	CGCACGCA TGTTGTACTTTTTTTT	CAGTGCGTGTCGTGGAGTC
hsa-miR-4286	CGCGCAACCCCACTCCT	CAGTGCGTGTCGTGGAGTC
hsa-miR-7974	CAAGGCTGTGATGCTCTCCT	CAGTGCGTGTCGTGGAGTC
hsa-miR-21-5p	CGCGCA TAGCTTATCAGACTGA	CAGTGCGTGTCGTGGAGTC
hsa-miR-27a-5p	CGCAAGGGCTTAGCTGCTTG	CAGTGCGTGTCGTGGAGTC
hsa-miR-92a-1-5p	CAAGGTTGGGATCGGTTGC	CAGTGCGTGTCGTGGAGTC
hsa-miR-101-3p	CCACACGCA TACAGTACTGTGATA	CAGTGCGTGTCGTGGAGTC
hsa-miR-221-5p	CACGCA ACCTGGCATACAATGT	CAGTGCGTGTCGTGGAGTC
hsa-miR-222-3p	CACGCA AGCTACATCTGGCTA	CAGTGCGTGTCGTGGAGTC
hsa-miR-424-3p	GCA CAAAACGTGAGGCGC	CAGTGCGTGTCGTGGAGTC
hsa-miR-424-5p	CGCGCACAGCAGCAATTCATGT	CAGTGCGTGTCGTGGAGTC
hsa-miR-3065-5p	GCGCGCATCAACAAAATCACTGAT	CAGTGCGTGTCGTGGAGTC
U6	AACGCTTCACGAATTTGCGT	CTCGCTTCGGCAGCACA

Sequence of shRNAs	
shMETTL3#1	TCAGTGGATCTGTTGTGAT
shMETTL3#2	AGGAACAATCCATTGTTGA
shMETTL3#3	TGCAAGTATGTTCACTATG

Sequence of miRNAs mimics	
miR-92b-3p mimic	UAUUGCACUCGUCCCGGCCUC

Primers for in vitro transcription	
Pri-miR-92b-Forward	CCGCTCGAGTAATACGACTCACTATAGGGAGAAGTCTGAGTACTTAAAGAGCAAGCGC
Pri-miR-92b-Reverse	CTAGTCTAGATAGAAGAGAAAGCCTGGGAGGGT
Pri-miR-1-1-Forward	CCGCTCGAGTAATACGACTCACTATAGGGAGAAAGGCTGTCCTGCTCACACA
Pri-miR-1-1-Reverse	CTAGTCTAGATCCCGGCCTGAGATACATAC

Primers for MeRIP-qPCR	
miR-92b-Forward	CTTCTGGGACTCCGCAAACT
miR-92b-Reverse	TTGGAGGCCAGAGAGACTTG

Probes for Northern blot	

Pri-miR-92b	GGAGGCCGGGACGAGUGCAAUA
Pri-miR-1-1	UCUUCCCUGGAGUCUACUGC
Pre-miR-92b	CAAUAUUGGCGGGGGAAAAA
Pre-miR-1-1	CUUAGCAGGUCCAUAUGGGC
miR-92b-3p	GGAGGCCGGGACGAGUGCAAUA

Materials and Methods

Cell lines and cell culture

Human embryonic kidney 293T (HEK293T) cells were obtained from the American Type Culture Collection (Manassas, VA, USA). NOZ cells were ordered from the Health Science Research Resources Bank (Osaka, Japan), and GBC-SD cells were purchased from the Cell Bank of Type Culture Collection of Chinese Academy of Sciences (Shanghai, China). All cell lines were cultured in Dulbecco's modified Eagle's medium (Gibco, USA), except that NOZ cells were cultured in William's E medium (Gibco, USA), all of them supplemented with 10% fetal bovine serum (Gibco, USA) with antibiotics (100 u/mL penicillin, 100 μg/mL streptomycin). All cell lines were authenticated free from mycoplasma by PCR.

Lenti-Pseudovirus production and transduction

HEK293T cells in 100-mm dishes were transfected with 4 μg of the required plasmids (overexpression constructs or shRNAs), 3 μg of psPAX and 2 μg of pMD2.G with 30 μl of polyethylenimine (PEI). Lentiviral supernatants were collected at 48 and 72 hours post-transfection and spun down at 300 g for 15 minutes to remove cell debris. Gallbladder cancer cells were plated into 60-mm dishes with medium containing virus supplemented with 8 μg/ml polybrene (Sigma), following positive selection with 2.5 μg/ml puromycin to generate stable cell lines.

Quantitative real-time PCR

Total RNA was extracted with TRIzol reagent (Sigma, USA) according to the manufacturer's instructions. cDNA was synthesized using the PrimerScript RT Reagent

Kit (Takara, China). The relative RNA levels were measured by qRT-PCR in triplicate using the Applied Biosystems ViiATM 7 Real-Time PCR system (Applied Biosystems, USA). β-Actin served as an internal control for mRNA quantification, while U6 small nuclear RNA was used in miRNA quantification. All the relative expression levels of RNAs were calculated with the $2^{-\Delta\Delta Ct}$ method.

Immunoblotting

Protein extracts from cells were prepared using RIPA lysis buffer (20 mM Tris, pH 7.4, 135 mM NaCl, 10% Glycerol, 1% NP-40, 0.5% NaDoc, 1 mM EDTA) supplemented with proteinase inhibitor and were quantified with the Micro BCA Protein Assay Kit (Thermo Fisher Scientific, USA). Total protein (20 μg) was subjected to 10% or 15% SDS-PAGE and then transferred to PVDF membrane (Millipore, Germany). Immunoreactive proteins were detected using the ECL Kit (Millipore, Germany).

MeRIP-qPCR

Total RNA were subjected to fragment (~ 100 nucleotide fragments) with Ambion fragmentation reagent (40 s incubation at 94 °C), then RNA were incubated with protein A beads previously bound anti-m6A polyclonal antibody (Synaptic Systems) or IgG (isotype control) in RIP buffer at 4 °C for 3 hours. Next, samples were washed as follows: RIP buffer for two time, low salt buffer (50 mM NaCl, 10 mM TRIS-HCL and 0.1% NP-40) for two times, high salt buffer (500 mM NaCl, 10 mM TRIS-HCL and 0.1% NP-40) for two times and once with RIP buffer. Finally, RNA were eluted by RLT buffer, followed by purification with Qiagen and subjected to qRT-PCR.

In vitro pri-miRNA processing assays

The pri-miR-92b was transcript *in vitro* by the T7 based MEGAshortscript kit (Life

Technologies). To obtain [m^{6}A] pri-miR-92b, ATP in the transcription reaction was instead by N6-methyl-ATP (m^{6}A) (Biorbit, orb65363). Briefly, pri-miR-92b or [m^{6}A] pri-miR-92b and pri-miR-1-1 (control) were incubated with lysates from 293T cells co-transfected with DGCR8 and DROSHA. The products were then purified and detected by northern blotting. In addition, the mutant pri-miR-92b, with A to T mutation at the m^{6}A site, was subjected to *in vitro* transcription, and followed by *in vitro* processing as described above. All primer sequences for *in vitro* transcription are shown in Supplementary Table S3.
